# CDK5RAP3, an essential regulator of checkpoint, interacts with RPL26 and maintains the stability of cell growth

**DOI:** 10.1111/cpr.13240

**Published:** 2022-05-04

**Authors:** Hongchen Yan, Jun‐jie Xu, Ilyas Ali, Wei Zhang, Ming Jiang, Guiping Li, Yong Teng, Guangxun Zhu, Yafei Cai

**Affiliations:** ^1^ College of Animal Science and Technology Nanjing Agricultural University Nanjing China; ^2^ Department of Stomatology, Tongji Hospital, Tongji Medical College Huazhong University of Science and Technology Wuhan China; ^3^ Department of Hematology and Medical Oncology, Winship Cancer Institute Emory University School of Medicine Atlanta Georgia USA

## Abstract

**Purpose and Materials:**

CDK5RAP3 (CDK5 regulatory subunit associated protein 3) was originally identified as a binding protein of CDK5. It is a crucial gene controlling biological functions, such as cell proliferation, apoptosis, invasion, and metastasis. Although previous studies have also shown that CDK5RAP3 is involved in a variety of signalling pathways, however, the mechanism of CDK5RAP3 remains largely undefined. This study utilized MEFs from conditional knockout mice to inhibit CDK5RAP3 and knockdown CDK5RAP3 in MCF7 to explore the role of CDK5RAP3 in cell growth, mitosis, and cell death.

**Results:**

CDK5RAP3 was found to be widely distributed throughout the centrosome, spindle, and endoplasmic reticulum, indicating that it is involved in regulating a variety of cellular activities. CDK5RAP3 deficiency resulted in instability of cell growth. CDK5RAP3 deficiency partly blocks the cell cycle in G_2_/M by downregulating CDK1 (Cyclin‐dependent kinase 1) and CCNB1 (Cyclin B1) expression levels. The cell proliferation rate was decreased, thereby slowing down the cell growth rate. Furthermore, the results showed that CDK5RAP3 interacts with RPL26 (ribosome protein L26) to regulate the mTOR pathway. CDK5RAP3 and RPL26 deficiency inhibited mTOR/p‐mTOR protein and induce autophagy, resulting in an upregulation of the percentage of apoptosis, and the upregulated percentage of apoptosis also slowed cell growth.

**Conclusions:**

Our experiments show that CDK5RAP3 interacts with RPL26 and maintains the stability of cell growth. It shows that CDK5RAP3 plays an important role in cell growth and can be used as the target of gene medicine.

## Graphical abstract

In normal, CDK5RAP3 is distributed in the centrosome, spindle and endoplasmic reticulum, the cells undergoes the growth and proliferation. However, when CDK5RAP3 is deficient, the cell cycle is blocked in G_2_/M and cell proliferation slows down, and the partial cycle block does not cause apoptosis. Additionally, CDK5RAP3 distributed in the endoplasmic reticulum combined with the deficiency of RPL26 will inhibit the mTOR pathway, aggravate autophagy and trigger apoptosis.

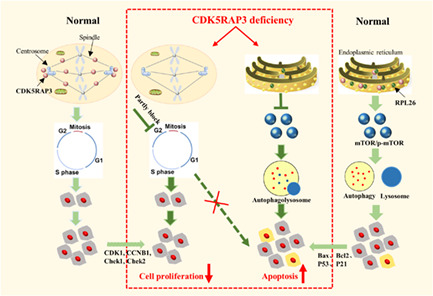

## 1 INTRODUCTION

CDK5RAP3 (CDK5 regulatory subunit associated protein 3, C53), also known as LZAP (LXXLL/leucine‐zipper‐containing ARF‐binding protein), IC53 (isoform of C53), HSF‐27(heat stress transcription factor 27), MST016, PP1553, OK/SW‐cl.114 (NCBI), was originally identified as a CDK5 binding protein two decades ago.^1^ CDK5RAP3 is involved in cell cycle regulation, cell damage, apoptosis, invasion, migration, metastasis, cytoskeletal remodelling, proliferation and other cellular processes.^2^, ^3^, ^4^, ^5^, ^6^, ^7^, ^8^, ^9^ It was found to regulate cell proliferation in a variety of cell lines, including ECV304 (an endothelial cell‐derived cell line), PLC/PRF/5 (liver cancer cell line), SMMC‐7721 (liver cancer cell line) and HepG2 (human hepatoma cell lines, HCC cell lines), gastric cancer cell lines (MKN74, AGS, MGC‐803, SGC‐7901, and HGC‐27), etc.^4^, ^7^, ^9^, ^10^ During embryonic development, CDK5RAP3 interacts with CBP (cAMP response element‐binding protein [CREB]‐binding protein), p25, and plays a key role in neuronal proliferation, migration and differentiation.^11^ CDK5RAP3 regulates the cell cycle through regulating with a number of cell cycle‐related proteins.^3^, ^12^, ^13^, ^14^ In CDK5RAP3‐depleted embryos, loss of CDK5RAP3 has been reported to result in G_2_/M arrest.^13^ According to Jiang's findings, overexpression of CDK5RAP3 reduced CDK1 (cyclin‐dependent kinase 1) phosphorylation and enhanced nuclear accumulation of CCNB1 (Cyclin B1).^3^ In 2009, Jiang's research found that knocking down CDK5RAP3 can delayed CDK1 activation and mitotic entry,^12^ and CDK5RAP3 interacts with Ufl1 (UFM1 specific ligase 1, KIAA0776, Maxer, NLBP, RCAD) to alter G_1_/S transition by decreasing CCND1 (Cyclin D1) transcription suppression through control of CDK5RAP3 nuclear translocation.^14^ Additionally, overexpression of CDK5RAP3, an important regulator of checkpoints, will partially counteract the activation of Chek1 (checkpoint kinases 1) and Chek2 (checkpoint kinases 2) in response to DNA damage, and CDK5RAP3 was an essential regulator of checkpoint.^12^

The CDK5RAP3 gene structure and protein sequence are highly conserved across species and are required for the formation of Zebrafish embryonic epithelial cell sheets.^13^, ^15^ Yang's team used a mouse knockout model and found that the CDK5RAP3 knockout mouse died at E16.5, indicating that CDK5RAP3 is required for liver development and function, as well as CDK5RAP3's role as a substrate adapter for the UFM1 (ubiquitin fold modifier 1) system.^16^, ^17^ According to research conducted by Michaela Quintero using an IEC (intestinal epithelial cell)‐specific knockout mouse model, CDK5RAP3 deficiency can lead to embryonic death, while CDK5RAP3 plays a key role in the growth and maintenance of Paneth cells.^18^

CDK5RAP3 expression has been linked to the occurrence, invasion, metastasis and invasion of a variety of malignancies.^5^, ^7^, ^8^, ^9^, ^19^, ^20^ Its expression was found to be highly correlated with the degree and depth of colon adenocarcinoma cell infiltration in the SW480 (a colon adenocarcinoma cell line).^21^ According to a number of studies, CDK5RAP3 plays a major role in controlling Wnt/β‐catenin, Akt and other signalling pathways in gastric cancer.^7^, ^15^, ^22^ Besides ARF (alternative reading frame), TUBG1 (γ‐tubulin), LHBs, Stat3, MCM6 (complex component 6) and Wip1 (wild‐type p53‐induced phosphatase 1, PPM1D) had also been linked to CDK5RAP3.^2^, ^6^, ^20^, ^24^, ^25^, ^26^, ^27^

CDK5RAP3 is overexpressed in human HCCs, lung adenocarcinoma and colon adenocarcinoma, and high levels of CDK5RAP3 activate PAK4 (p21‐activated protein kinase 4) to enhance HCC metastasis.^4^, ^21^, ^29^ However, in gastric tumour tissues and renal cancer tissues, the CDK5RAP3 expression of mRNA and protein levels was lower as compared with surrounding non‐tumour tissues.^7^, ^8^, ^22^, ^23^ Whether CDK5RAP3 is a tumour activator or an inhibitor depends on the amount of expression in different cancer tumours.

Because CDK5RAP3 interacts with and anchors to ER (endoplasmic reticulum) membrane protein in Ufl1 and anchors to the ER, CDK5RAP3 may be an ER protein as well.^14^ CDK5RAP3 appears to be involved in the ER‐stress response in plants.^29^ The ER homoeostasis is maintained by the Ufm1 system.^30^, ^31^, ^32^ CDK5RAP3 interacts with numerous components of the Ufm1 conjugation system (such as Ufm1, Ufl1, Ufbp1 and DDRGK1), according to several studies.^23^, ^29^, ^30^, ^33^ RPL26 (a ribosome protein L26) is a biological target of UFMylation and has been reported to serve as a functional link between UFMylation and ER protein homoeostasis by facilitating the translocation of co‐translated proteins to the ER.^34^, ^35^, ^36^ Despite its possible role in biological function and critical signalling networks, the physiological role of CDK5RAP3 are unknown. In this research, the conditional knockdown CDK5RAP3 gene cell line MEFs and lentivirus were used to stably transfect the CDK5RAP3 gene knockdown cell line MCF7 for biological research. After CDK5RAP3 deficiency, cell proliferation and apoptosis have an impact on cell growth. CDK5RAP3 is mainly located to centrosome and spindle during mitosis, and CDK5RAP3 deficiency reduces the stability of cell growth by partially blocking the cell cycle in G_2_/M. Apoptosis was not induced by partially blocking the cell cycle in G_2_/M. The apoptosis was regulated by CDK5RAP3 interacting with RPL26 located in the endoplasmic reticulum, thereby inhibiting the mTOR pathway.

## 2 MATERIALS AND METHODS

### 2.1 Cell culture

CDK5RAP3^F/F^: CAG‐CreERT2 mice were used to create immortalized MEFs. Harvest mouse embryos at E13 or E14 (genotyping, CDK5RAP3^F/F^: CAG‐CreERT2/ERT2^+/+^ or CDK5RAP3^F/F^: CAG‐CreERT2^+/−^). Tissue from the trunk was collected and washed in PBS. Minced and added 1 ml of 0.05% trypsin/EDTA to each piece (Gibco, NY, USA). Dissociate cells by pipetting up and down thoroughly after 5 min of incubation. In DMEM basal medium, the left cells that can attach to the flask are fibroblasts (HyClone, Utah, USA). After 24 h, the cells should be 80%–90% confluent, then transfect with T lentivirus, select the positive immortalized cells with puromycin (10 μg/ml, InvioGen, ant‐pr‐1, USA) after 2 days, and culture the positive cells in new dishes. MEFs, MCF7 and 293FT cells were grown at 37°C with 5% CO_2_ in DMEM basic medium supplemented with 10% fetal bovine serum (Gibco, USA) and 1% penicillin/streptomycin (Solarbio, Beijing, China). When the cells were subcultured, the seeding density was 10^5^ in 60 mm dishes. To generate CDK5RAP3 deficiency, MEFs were treated for 4 days with 4‐OHT (4‐hydroxytamoxifen, 2 μM, H7904 or H6278, Sigma, USA) and ethanol (EtOH) as a control group. Nocodazole (100 ng/ml, Abcam, ab120630, USA) was added to the appropriate medium for 3 h prior to collection of MEFs. Starvation was induced using serum‐free medium for 6 h prior to sample collection. MEFs were given rapamycin (200 nM, Selleck, S1039) for 24 h to induce autophagy.

### 2.2 Plasmid transfection

Plasmids (LP1, LP2, PVSVG, CDK5RAP3 shRNA[recorded below as shRNA, CDK5RAP3 shRNA: GGCAGGAGAUUAUAGCUCU], and Scrab) were inserted into XL1‐Blue (Biomed, China), plate it in a solid medium and supplemented with ampicillin (Amp). Plasmid DNA was extracted using the E.Z.N.A.® Endo‐free Plasmid DNA Mini Kit II (OMEGA, D6950‐01, USA) to extract plasmid DNA according to the instructions. In Opti‐MEM medium (Gibco,USA), 10 μg plasmid (LP1: LP2: PVSVG: shRNA/Scrab = 3:2:3:3), 10 μl lipofectamine 2000 (Invitrogen, 13778, USA), and 10 μg/ml Polybrene (Solarbio, H8761) were incubated for 30 min. Add the mix into a six‐well plate, and add 2 ml of PS‐free medium with 1.5 × 10^6^ 293FT cells. Incubate for an additional 24–30 h and change the medium with the basic medium. After 24 h, the cells and other residues were filtered out to obtain the virus‐containing stock solution. The stock solution was added to the medium (1:1) to transfect MCF7 for 24 h, and then puromycin was added for the next selection. Stable transfection of CDK5RAP3 knockdown MCF7 (shRNA) and Scrab transfection control (Scrab) group were generated.

### 2.3 Immunofluorescence staining

The cells were collected and fixed for 15 min in 4% paraformaldehyde. Then permeabilized with PBST (0.5% Triton X‐100 in PBS) for 10 min before being blocked with PBST containing 10% normal goat serum for 30–45 min at room temperature. The primary antibodies were then treated for 45–60 min at 37°C with TUBG1, β‐tubulin, CDK5RAP3, LC3B, RPL13 and RPL26 (details in Table S1). The cells were then rinsed in PBS (containing 1% BSA) and incubated at 37°C with CY3‐conjugated goat anti‐rabbit and FITC‐conjugated goat anti‐mouse (details in Table S1). Finally, the cells were stained with Hoechst 33342 for 5 min after removing the redundant second antibody and washing with PBS for 5 min. A confocal microscope (Zeiss LSM 900 META, Jena, Germany) was used for photography.

### 2.4 RNA extraction and RT‐qPCR

The total RNA was extracted from MEFs and MCF7 with the RNA prep pure Micro Kit (Aidlab, RN07, Beijing, China). TransScript One‐Step gDNA Removal and cDNA Synthesis SuperMix were used to make the cDNA (TransGen, AT311‐03, Beijing, China). QuantStudio 5 was used to perform RT‐qPCR amplification using specified primers (Table S2) (Applied Biosystems, CA, USA). The relative transcript abundance was determined using the 2^−ΔΔCt^ method and normalized using the housekeeping gene actin as a reference.

### 2.5 Live cell staining of lysosomes

Take 1 μl Lyso‐Tracker Red (Beyotime, C1046, Beijing, China) and add it to 20 ml cell culture medium or an appropriate solution (such as PBS or HBSS containing calcium and magnesium ions). After mixing, pre‐incubate the cells at 37°C for 15 min. Remove the staining working solution and add a fresh cell culture solution. Bright and intense fluorescent staining of lysosomes was observed and photographed with a confocal laser microscope at 590 nm.

### 2.6 Western blot analysis

Proteins were extracted with RIPA lysis buffer (Beyotime, P0013C, Nantong, China) containing Cocktail (Beyotime, P1081, China) and PMSF (Beyotime, ST506, China) by lysing cells. Then 5× SDS (Beyotime, P0285, China) was mixed with the samples and boiled for 5 min at 100°C for proteins denaturation. The SDS–PAGE was used to separate proteins. The proteins were transferred onto 0.2 μm poly‐vinylidene fluoride (PVDF) membrane (Millipore, ISEQ00010, USA) and blocked in TBST containing 5% skimmed milk powder for 2–4 h. The membranes were incubated with primary antibody (Table S1) at 4°C, and then horseradish peroxidase (HRP)‐conjugated corresponding secondary antibody (Table S1) was incubated at room temperature for 60–90 min. Ultimately, the BeyoECL Plus Kit (Beyotime, P0018FS, China) was used for ChemiDoc MP (bio‐rad). The relative expression level of the target protein to actin was calculated by the software AlphaView SA (ProteinSimple, CA, USA) and the normalization method.

### 2.7 COIP

The r‐protein A/G magenetc IP/Co‐IP kit (ACE, China) was used to process the samples. Collect the cells in a centrifuge tube, add 0.1% lysis/washing buffer enhanced, poke the cells with the tip of a 1 ml syringe, incubate on ice for 30 min and centrifuge at 13,000 *g* to remove the precipitation. Add the primary antibodies CDK5RAP3 and IgG as control, and incubate it overnight at 4°C. Pipette r‐protein A/G magpoly beads and wash with lysis/washing Buffer, then add the above‐mentioned lysis buffer containing the primary antibody, and incubate at room temperature for 2 h. Finally, remove the magnetic beads and add 5 × SDS and boil it at 95–100°C for 10 min. The next steps were followed the WB operation.

### 2.8 RNA interference

The siRNA‐targeting RPL13 (siRPL13‐1, siRPL13‐2, siRPL13‐3), RPL26 (siRPL26‐1, siRPL26‐2, siRPL26‐3), and non‐sense interfering RNA (non‐sense control, NC) were purchased from GenePharma Co., Ltd. (Shanghai, China) (Table S3). Each siRNA was transfected at a concentration of 30 pmol/L into the cells using lipofectamine 2000 (11668; Invitrogen, Carlsbad, CA) as the manufacturer's protocol. The MEFs was collected 72 h after transfection for further experiments.

### 2.9 Kits related

Crystal violet (Macklin, 548‐62‐9, Shanghai, China) was used for crystal violet staining. 5‐Ethynyl‐20‐deoxyuridine (EDU) (100 mM) (BeyoClickTM EdU‐555, C0075S, China) was used for detecting the percent of cell proliferation. Annexin V‐FITC/PI apoptosis detection kit (Vazyme, A211, China) was performed, according to the manufacturer's instruction. Cell cycle detection kit (Keygentec, KGA511, China) was used for detecting cell cycle. The JC‐1 apoptosis assay kit (Keygentec, KGA601, China) was used according to the kit protocol. Reactive oxygen species assay kit (Beyotime, S0033, China) was used to detect the ROS level of cells. The r‐protein A/G magenetc IP/Co‐IP kit (ACE, China) was used to Process the COIP samples. For more detailed steps refer to the attached Supplementary Methods and Materials.

### 2.10 Statistical analysis

Data analysis was performed using GraphPad Prism 8 (GraphPad Software, San Diego, CA) with One‐way analysis of variance (ANOVA) followed by Tukey's test for multiple comparisons. *p* > 0.05 means not significant (ns); *p* < 0.05 means statistically significant difference (*); *p* < 0.01 means highly significant difference (**); *p* < 0.001 means extremely significant difference (***). The data were expressed as mean ± standard error (SD) and all the experiments were repeated at least three times.

## 3 RESULTS

### 3.1 CDK5RAP3 deficiency slow down cell growth

**FIGURE 1:**
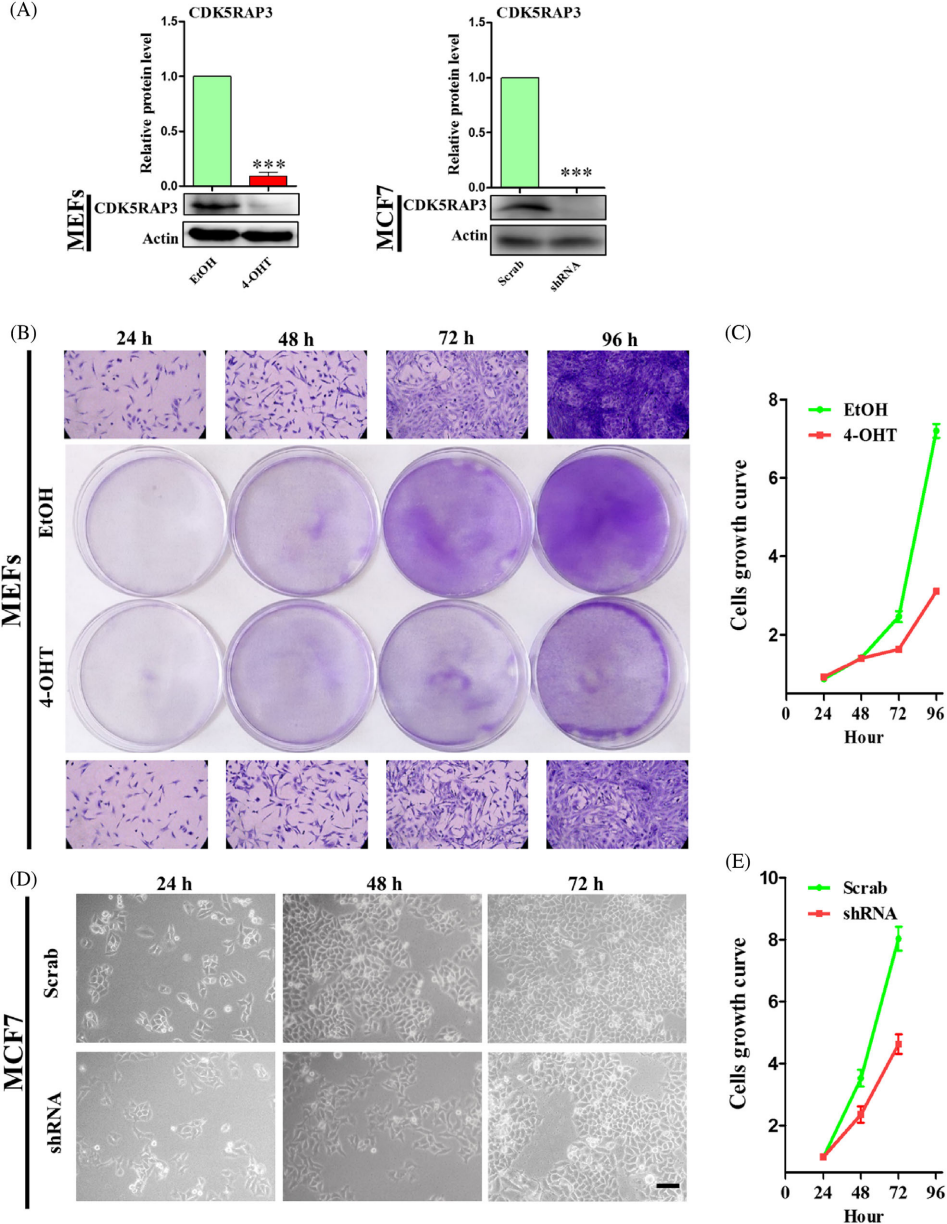
The cell growth of conditional knockout CDK5RAP3 in MEFs and lentivirus stable conversion CDK5RAP3 in MCF7. (A) CDK5RAP3 protein level in MEFs treated with EtOH (control) and 4‐OHT for 4d (left); CDK5RAP3 protein level of MCF7 transfected with Scrab (control) and CDK5RAP3 shRNA (shRNA). (B) Crystal violet staining for 24, 48, 72 and 96 h treated with EtOH and 4‐OHT in MEFs. (C) Spectrophotometer detects the absorbance at 590 nm and draws the growth curve for 24, 48, 72 and 96 h treated with EtOH and 4‐OHT in MEFs. (D) Observe the morphology of MCF7 cells transfected with Scrab and shRNA under light microscope in 24, 48 and 72 h. Scale bar = 100 μm. (E) Calculate the number of cell growth per unit area at 24, 48 and 72 h to draw a growth curve in MCF7 transfected with Scrab and shRNA. The results are presented as mean ± SD. *p < 0.05; **p < 0.01

Immortalized MEFs generated from CDK5RAP3^F/F^: CAG‐CreERT2 mice were treated with 4‐hydroxytamoxifen (4‐OHT) to induce CDK5RAP3 deficiency to explore its function. Compared with the EtOH (control) group, the protein level of CDK5RAP3 decreased by more than 90% after 4 days of 4‐OHT treatment, as shown in Figure 1A. In addition, the protein level of CDK5RAP3 was down‐regulated in 57 kDa in the shRNA group compared with Scrab (control) group (Figure 1A). Microscopically, in MEFs and MCF7, the number of cells lacking CDK5RAP3 in logarithmic growth phase was sparse, for which cell growth curves were measured. The results of crystal violet staining showed that there was no significant change in cells number at 24–48 h after 4‐OHT treatment, however, the growth of the cells number was slower than the EtOH group, as shown in Figure 1B,C. MCF7 developed from a single suspension state at the time of inoculation to an adherent state after 24 h, with no significant difference in cell growth state between the Scrab and shRNA groups (Figure 1D). However, at 24–48 h, the cell growth density in the shRNA group was considerably lower than in the Scrab group. In conclusion, the CDK5RAP3 deficiency delays cell growth.

### 3.2 CDK5RAP3 deficiency reduce EDU positive cell number

**FIGURE 2:**
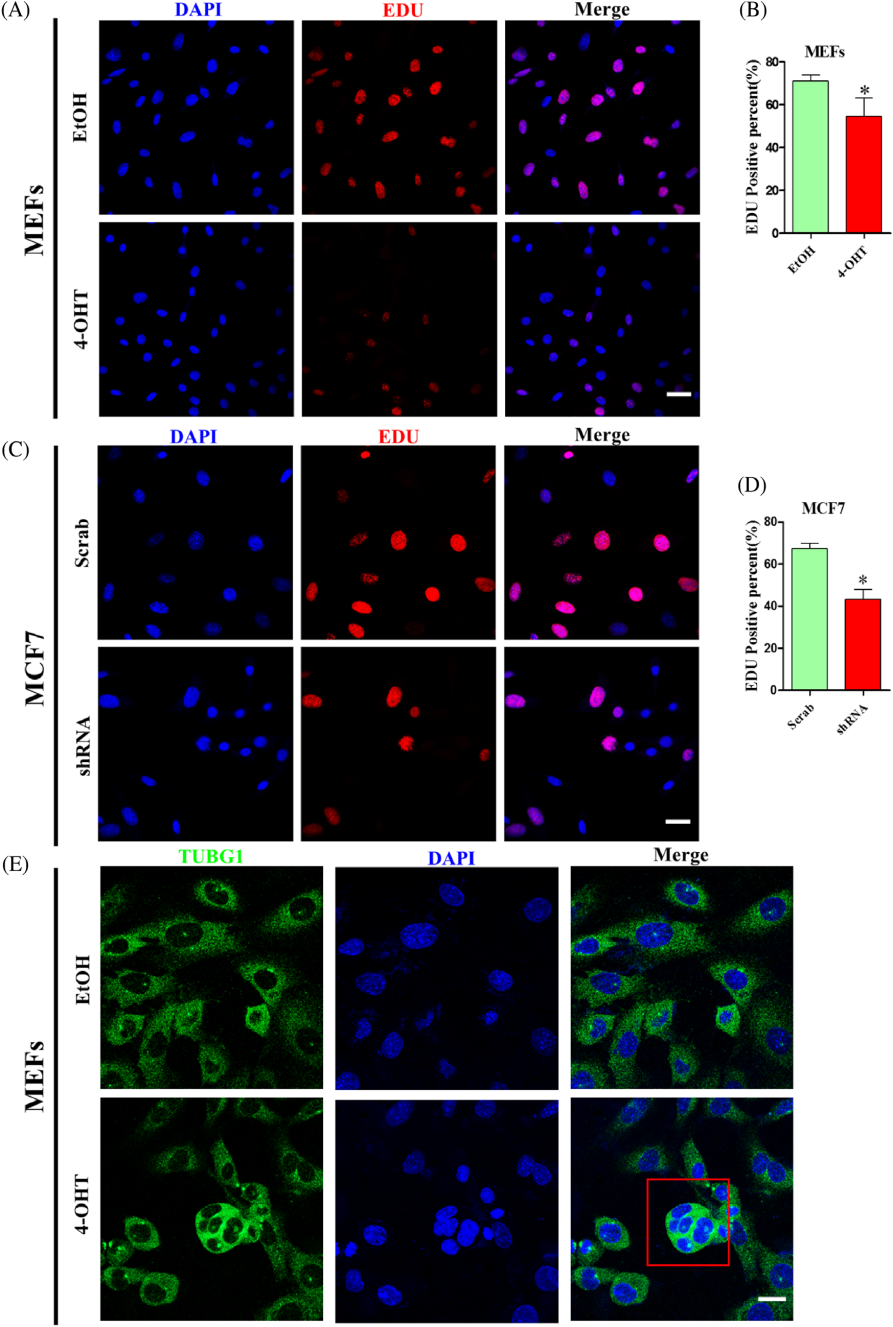
CDK5RAP3 deficiency reduce EDU positive cell number. (A) EDU immunofluorescent staining (CY3) in MEFs treated with EtOH and 4‐OHT. Scale bar = 40 μm. (B) Percentage statistics of EDU positive cell number in EtOH group and 4‐OHT group. (C) EDU immunofluorescent staining (CY3) in Scrab group and shRNA group. Scale bar = 40 μm. (D) Percentage statistics of EDU positive cell number in Scrab group and shRNA group. (E) Cellular immunofluorescence staining for cytoskeleton protein TUBG1 in MEFs. Scale bar = 10 μm. The results are presented as mean ± SD. *p < 0.05; **p < 0.01

Changes in cell growth and cell numbers are usually affected by cell proliferation or apoptosis. Therefore, we used EDU‐labelled staining to test whether proliferation was affected. After MEFs were treated with 4‐OHT for 4 days, the percentage of EDU‐positive cells was calculated. As Figure 2A,B indicates, the percentage of EDU‐positive cells was down‐regulated by 13% compared with the EtOH group. Similarly, the percentage of EDU‐positive cells was calculated in MCF7. As Figure 2C,D indicates, the percentage of EDU‐positive cells was down‐regulated by 20% compared with the Scrab group. Hence these results testify that CDK5RAP3 deficiency reduces cell proliferation. Simultaneously, the results of immunofluorescence staining of TUBG1 for Cytoskeleton showed abnormal multinucleation (Figure 2E) in MEFs, which may indicate abnormal mitosis after CDK5RAP3 deficiency.

### 3.3 CDK5RAP3 deficiency leads to an increase in cell cycle arrest at G_2_ /M

**FIGURE 3:**
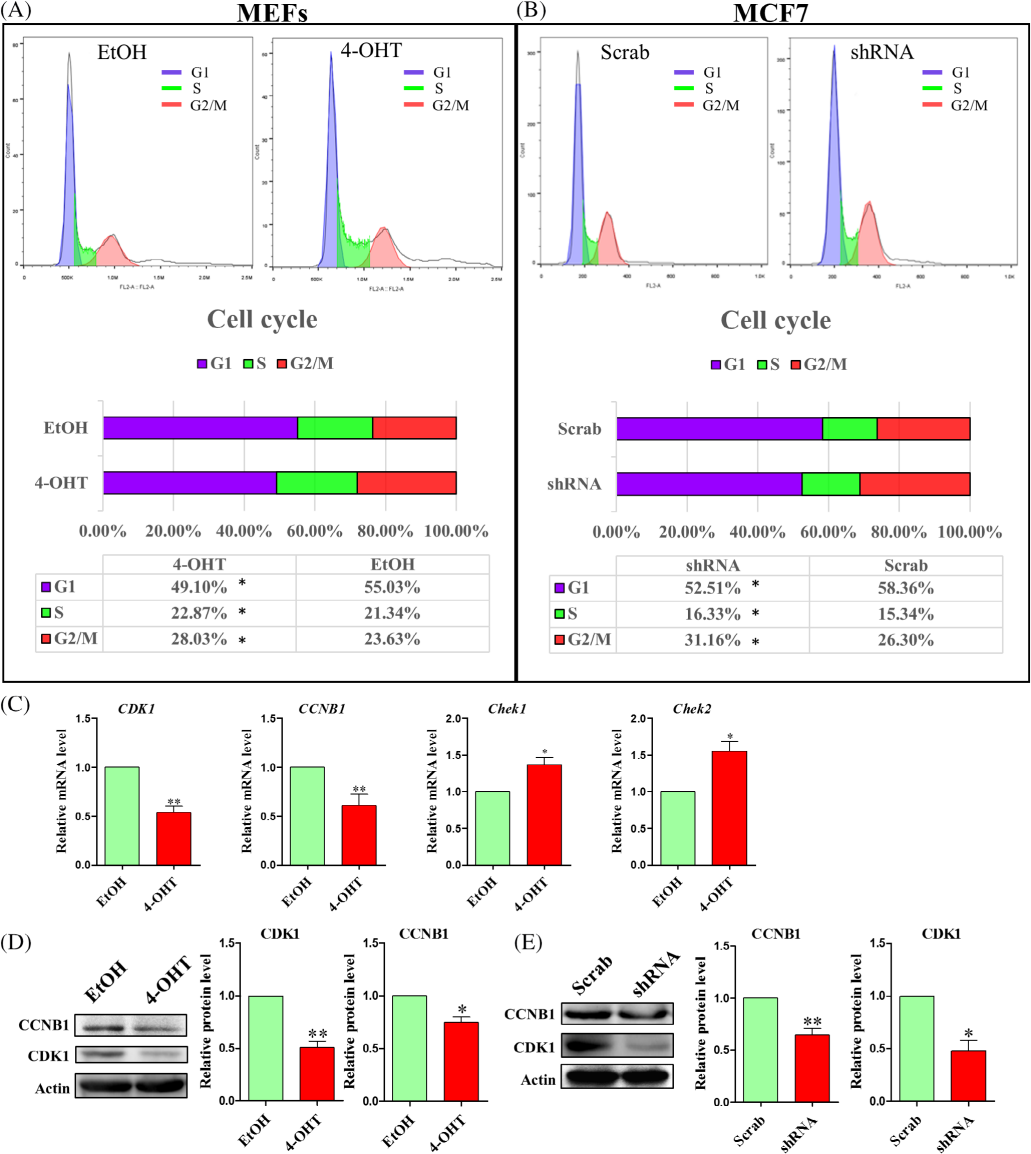
Effects of CDK5RAP3 deficiency on the cell cycle progression. (A) Flow cytometry analysis of cell cycle in EtOH group and 4‐OHT group. (B) Flow cytometry analysis of cell cycle in Scrab group and shRNA group. (C) Relative mRNA expression level of *CDK1, CCNB1, Chek1* and *Chek2* in EtOH group and 4‐OHT group. (D) Representative WB and relative densitometry analysis of CDK1 and CCNB1 in EtOH group and 4‐OHT group. (E) Representative WB and relative densitometric analysis of CDK1 and CCNB1 in Scrab group and shRNA group. The results are presented as mean ± SD. *p < 0.05; **p < 0.01

An increase or decrease in cell proliferation is often accompanied by abnormal mitosis during the cell cycle.^12^, ^37^, ^38^ Therefore, we performed flow cytometry to investigate the effect of CDK5RAP3 deficiency on the cell cycle distribution of MEF and MCF7. As shown in Figure 3A, CDK5RAP3 deficiency significantly reduced the proportion of MEFs in G_0_/G_1_ phase (55.03 ± 0.79% vs. 49.10 ± 1.12%, *p* < 0.01) and increased in S phase (21.34 ± 0.54% vs. 22.87 ± 0.37%, *p* < 0.01), the proportion of MEFs in G_2_/M phase was markedly increased after treatment with 4‐OHT (23.63 ± 1.21% vs. 28.03 ± 1.57%, *p* < 0.01). Similarly, CDK5RAP3 deficiency (shRNA group) significantly reduced the proportion of MCF7 in G_0_/G_1_ phase (58.36 ± 1.51% vs. 52.51 ± 1.37%, *p* < 0.01; Figure 3B) and increased in S phase (15.34 ± 0.77% vs. 16.33 ± 0.39%, *p* < 0.01; Figure 3B), the proportion of MCF7 in G_2_/M phase was markedly higher compared with Scrab group (26.30 ± 0.93% vs. 31.16 ± 1.24%, *p* < 0.01; Figure 3B).These results indicate that the growth promotion observed after CDK5RAP3 deficiency might be associated with cell cycle arrest at G_2_/M (Figure 3B).

Additionally, the cell cycle‐related genes CDK1 and CCNB1 were elevated in MEFs (Figure 3C). In contrast, the mRNA expression levels of Chek1 and Chek2 were increased (Figure 3C). Additionally, the protein expression levels of CDK1 and CCNB1 were decreased in both MEFs and MCF7 after CDK5RAP3 deficiency (Figure 3D,E). Taken together, CDK5RAP3 deficiency down‐regulates CDK1 and CCNB1 and leads to an increase in cell cycle arrest at G_2_/M.

### 3.4 A portion of CDK5RAP3 is localized at the centrosome and spindle

**FIGURE 4:**
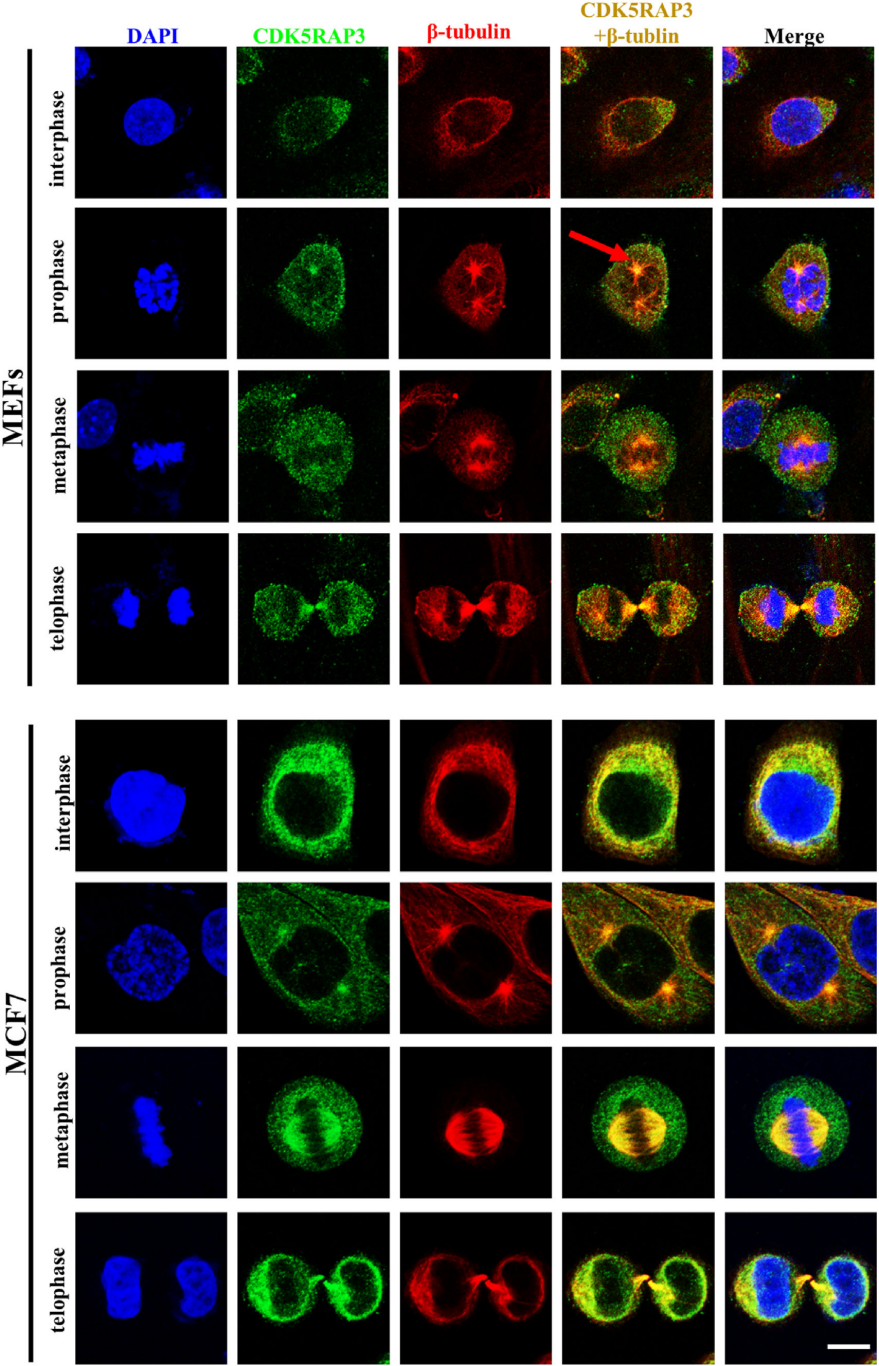
Dynamic expression of CDK5RAP3 protein in mitosis. The immunofluorescence with CDK5RAP3‐FITC, β‐tubulin‐CY3 (a microtubule protein) and Hoechst 33342 (blue) of normal MEFs (up) and MCF7 (down) in interphase, prophase, metaphase and telophase. Scale bar = 10 μm

In order to understand the role of CDK5RAP3 in G_2_/M, we explored the distribution of CDK5RAP3 in mitosis, we collected normal MEFs and MCF7 at different cell cycles stages (interphase, prophase, metaphase and telophase) and analysed β‐tubulin (located at microtubule) and the target protein CDK5RAP3 in MEFs and MCF7s. As shown in Figure 4, the cells in the interphase are mainly located in the cytoplasm, and CDK5RAP3 is mainly located in the cytoplasm, and a portion of proteins is located on the centrosome besides cytoplasm in prophase, and in metaphase, part of the CDK5RAP3 protein is combined with the spindle. At telophase, it is located on the cytoplasm and spindle where cell division occurs. CDK5RAP3 is partly located in the centrosome and partly changes with the change of spindle migration, suggesting that CDK5RAP3 plays an important role in mitosis as a centrosome and spindle‐associated protein.

### 3.5 CDK5RAP3 deficiency promoted apoptosis

**FIGURE 5:**
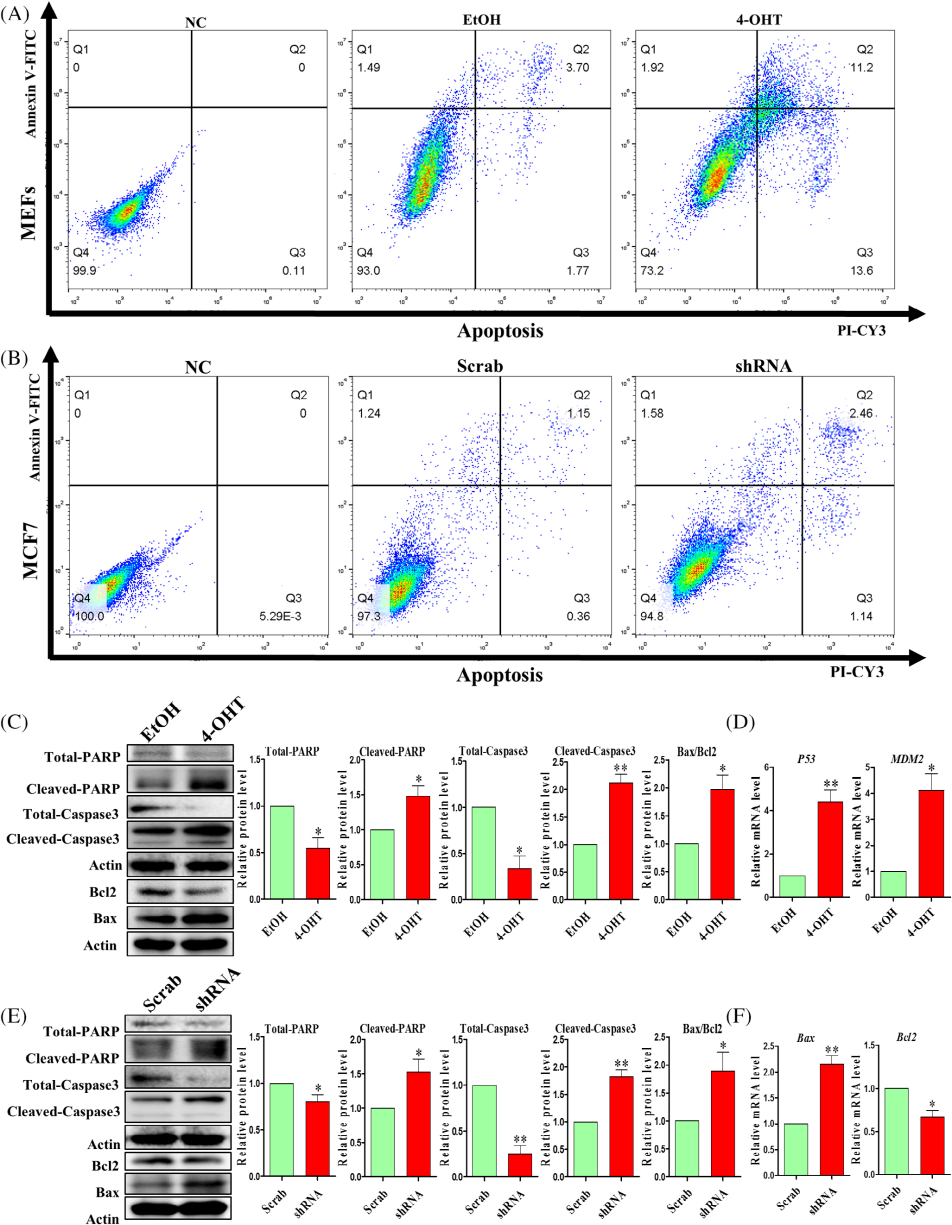
The effect of CDK5RAP3 deficiency on apoptosis. (A) Flow cytometry analysis of apoptosis in EtOH group and 4‐OHT group. (B) Flow cytometry analysis of apoptosis in Scrab group and shRNA group. (C) Representative WB and relative densitometry analysis of total‐PARP, cleaved‐PARP, total‐caspase3, cleaved‐caspase3, Bax and Bcl2 in EtOH group and 4‐OHT group. (D) Relative mRNA expression level of *P53 and MDM2* in EtOH group and 4‐OHT group. (E) Representative WB and relative densitometry analysis of total‐PARP, cleaved‐PARP, total‐caspase3, cleaved‐caspase3, Bax and Bcl2 in Scrab group and shRNA group. (F) Relative mRNA expression level of *Bax and Bcl2* in EtOH group and 4‐OHT group. The results are presented as mean ± SD. *p < 0.05; **p < 0.01

Changes in cell number and growth may also be related to the occurrence of cell apoptosis.^39^ Annexin V‐FITC and PI‐CY3 were used to detect cell apoptosis by flow cytometry. As the results shows in Figure 5A,B, that CDK5RAP3 deficiency caused a 19.8% reduction in live cells in MEFs (93 ± 1.56% vs. 73.2 ± 1.72%, *p* < 0.01; Figure 5A) and 2.5% reduction in live cells in MCF7 (97.3 ± 0.42% vs. 94.8 ± 0.51%, *p* < 0.01; Figure 5B). Subsequently, the percentage of apoptosis was increased in MEFs and MCF7 after CDK5RAP3 deficiency (Figure 5A,B). The DNA repair‐related protein PARP, total‐caspase3 and anti‐apoptotic protein Bcl2 were down‐regulated (Figure 5C). Furthermore, the protein levels of cleaved‐PARP, pro‐apoptotic proteins cleaved‐caspase3, and Bax were remarkably up‐regulated in MEFs (Figure 5C). A subset of pro‐apoptotic genes, including *P53* and *MDM2*, were also up‐regulated (Figure 5D). Similar to MEFs, the protein expression levels of PARP, total‐caspase3 and Bcl2 were down‐regulated (Figure 5E) in MCF7. The protein expression levels of cleaved‐PARP, cleaved‐capase3 and Bax were remarkably up‐regulated in MCF7 (Figure 5E). Besides, the mRNA expression levels of *Bax* and *Bcl2* were similar to the proteins expression (Figure 5E,F).

In addition, JC‐1 and ROS kits were used to detect changes in cell mitochondrial membrane potential and oxidative stress. In MEFs, most JC‐1 exists in the form of FITC^−^/CY3^+^ (Q1) and FITC^+^/CY3^+^ (Q2) in the EtOH group (Figure S1A), but JC1 exists mainly in the form of FITC^+^/CY3^+^ (Q2) and FITC^+^/CY3^−^ (Q3) in the 4‐OHT group (Figure S1A). In MCF7, JC1 exists in the form of FITC^−^/CY3^+^ (Q1) and FITC^+^/CY3^+^ (Q2) in the Scrab group (Figure S1C), but JC1 exists in the form of FITC^+^/CY3^+^ (Q2) was increased compared with the shRNA group (Figure S1C). It suggested that the intensity of red (CY3^+^) light was weakened, and cells undergone apoptosis. Moreover, the results of flow cytometry showed an increase in ROS levels in MEF and MCF7 after CDK5RAP3 deficiency (Figure S1B,D). Above all, CDK5RAP3 deficiency affects mitochondrial function and ROS levels and promotes apoptosis.

### 3.6 Apoptosis may not be due to Cell cycle arrest in G_2_ /M phase

**FIGURE 6:**
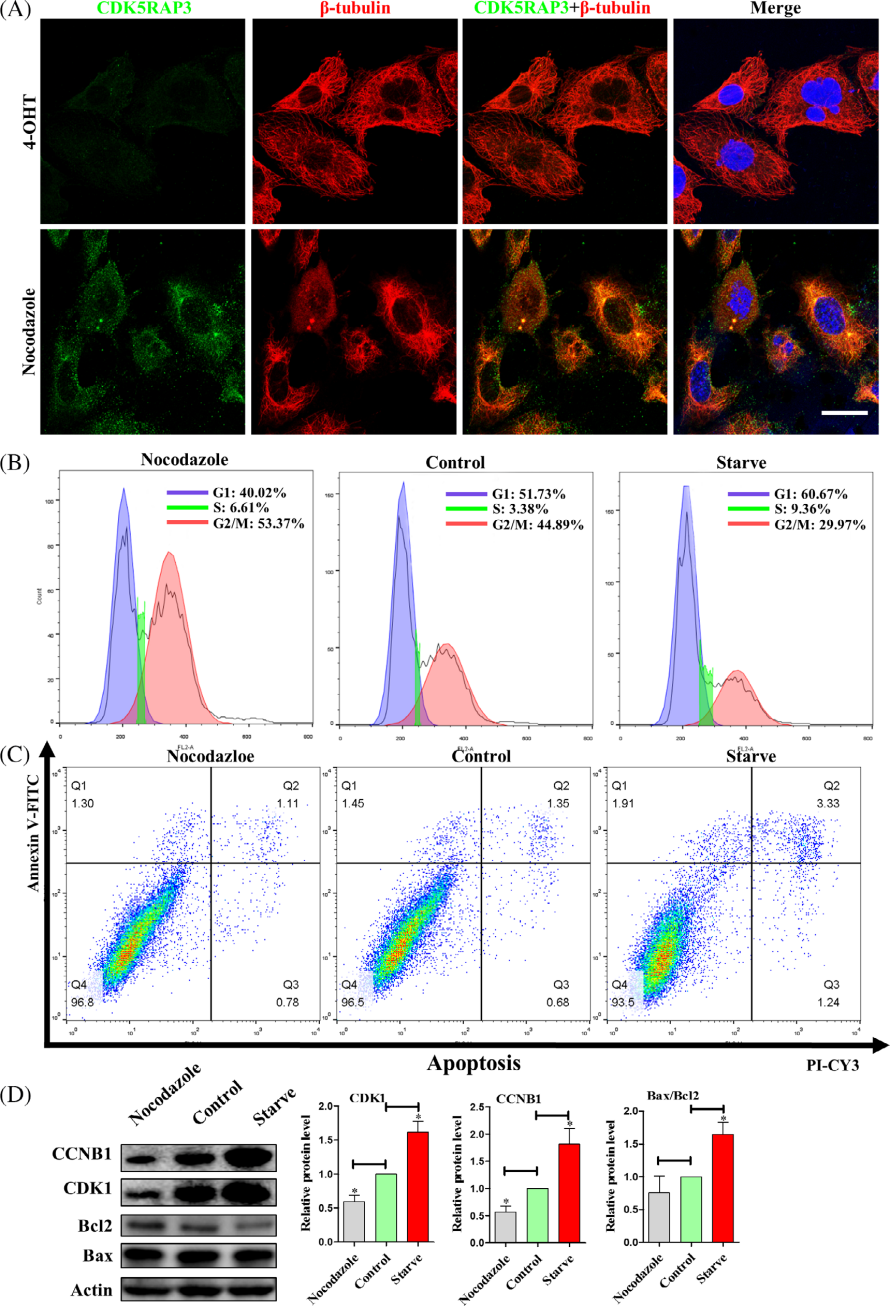
Cell cycle arrest in G2/M phase does not lead to apoptosis. (A) The immunofluorescence with CDK5RAP3‐FITC and β‐tubulin‐CY3 in EtOH and nocodazole blocked for 3 h.Scale bar = 10 μm. (B) Flow cytometry analysis of cell cycle in nocodazole blocked for 3 h, control and serum‐starved (Starve) for 6 h. (C) Flow cytometry analysis of apoptosis in nocodazole for 3 h, control and starve for 6 h. (D) Representative WB and relative densitometry analysis of CDK1, CCNB1, Bax and Bcl2 in nocodazole for 3 h, control and starve for 6 h. The results are presented as mean ± SD. *p < 0.05; **p < 0.01

From the results above, we have known that CDK5RAP3 could be as a centrosome and spindle binding protein, and CDK5RAP3 deficiency leads to cell cycle partly arrest in the G_2_/M phase. On the other hand, CDK5RAP3 deficiency promotes apoptosis. Is there a direct relationship between the partly cell cycle arrest caused by the loss of CDK5RAP3 and the occurrence of apoptosis? We used nocodazole to destroy the cell spindle disaggregation and block the cell cycle at G_2_/M, and serum starvation was used to block the cell cycle at G_0_/G_1_. Immunofluorescence staining of CDK5RAP3 and β‐tubulin in 4‐OHT and nocodazole blocked for 3 h groups (Figure 6A). The results showed that the CDK5RAP3 protein of nocodazole‐treated cells was still located on the centrosome, and CDK5RAP3 might be an upstream protein of microtubulin.

Flow cytometry analysis as shown in Figure 6B, the percent cells in the G_2_/M was increased 6.2% compared with the control group after nocodazole‐treated MEFs for 3 h, and the percent cells in G_2_/M was decreased 14.1% compared with the control group after MEFs were serum starved for 6 h. The protein expression levels of CDK1 and CCNB1 were significantly up‐regulated after nocodazole was blocked for 3 h and significantly down‐regulated after serum starvation for 6 h (Figure 6D). After 3 h of nocodazole treatment, the degree of cell cycle arrest in G_2_/M phase was greater than the partial cell cycle arrest caused by CDK5RAP3 deficiency (14.1% vs. 5.4%, Figures 3A and 6A). Then we perform flow cytometry to detect the percentage of apoptosis. As results are shown in Figure 6B, after 3 h of MEF treatment with nocodazole, there was almost no significant change in viable cells (96.5 ± 0.32% vs. 96.8 ± 0.27%). However, after 3 h of serum starvation, live cells (96.5 ± 0.32% vs. 93.5 ± 0.21%) decreased by 3.0%. Consistented with to above results, 3 h after nocodazole was blocked, the protein levels of Bax and Bcl2 were hardly affected (Figure 6C). However, after 6 h of serum starvation, the protein level of Bax increased and Bcl2 decreased significantly (Figure 6C). Therefore, the apoptosis might not be due to partial cell cycle arrest in the G_2_/M phase after CDK5RAP3 deficiency.

### 3.7 CDK5RAP3 deficiency regulated mTOR pathway and lead autophagy

**FIGURE 7:**
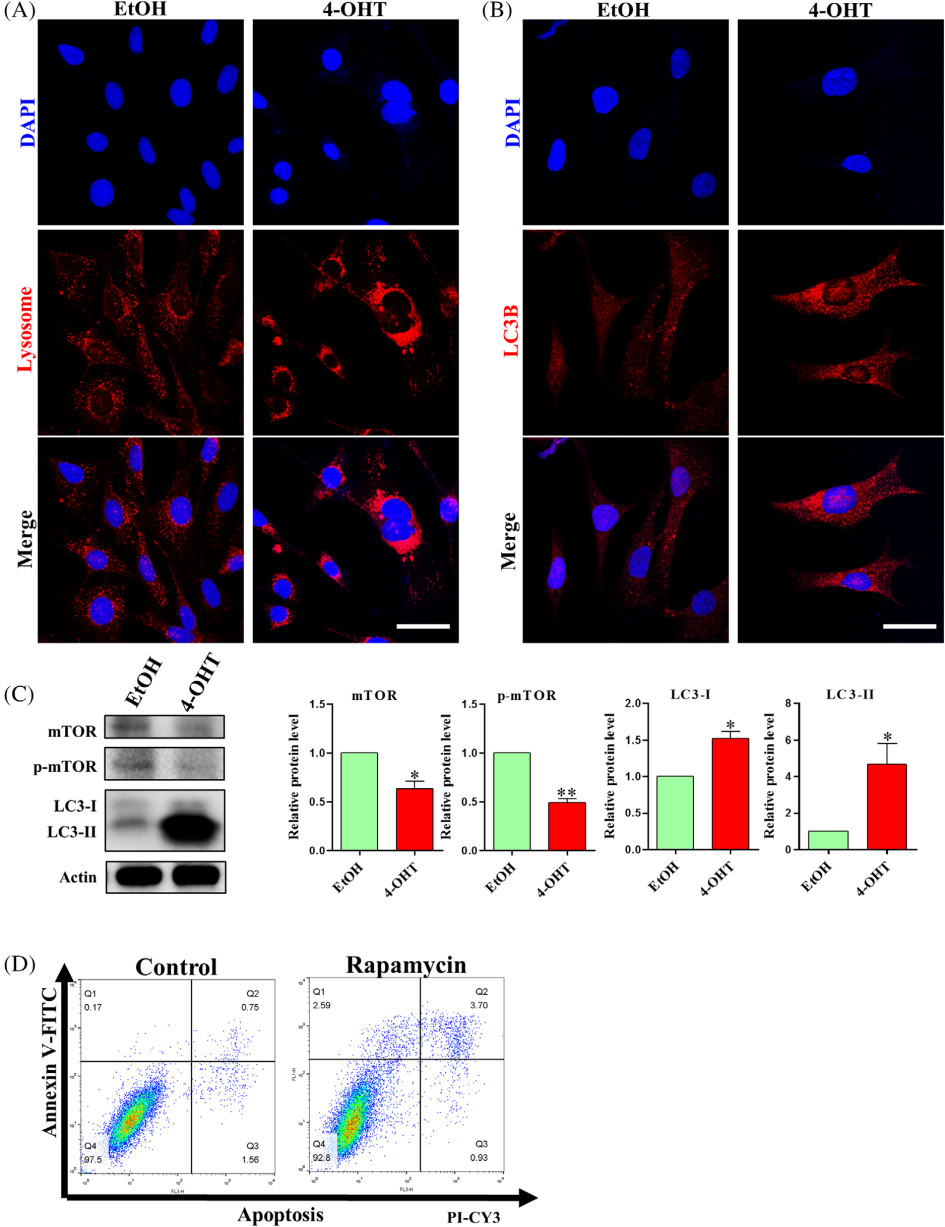
CDK5RAP3 deficiency causes autophagy to be regulated by mTOR pathway. (A) Lysosome‐tracker stains lysosomes in EtOH group and 4‐OHT group. Scale bar = 10 μm. (B) Immunofluorescence staining of LC3B in EtOH group and 4‐OHT group. Scale bar = 10 μm. (C) Representative WB and relative densitometry analysis of mTOR, p‐mTOR, LC3‐I and LC3‐II in EtOH group and 4‐OHT group. (D) Flow cytometry analysis of apoptosis in control and rapamycin treated for 24 h. The results are presented as mean ± SD. *p < 0.05; **p < 0.01

Double immunofluorescence staining for CDK5RAP3 and calnexin (a calcium‐binding protein located on the endoplasmic reticulum membrane) showed that part of the CDK5RAP3 protein was located in the endoplasmic reticulum (Figure S3A). There are multiple inseparable links between apoptosis and autophagy.^40^, ^41^ Lyso‐Tracker Red was used to stain lysosomes in living cells. As results shown in Figure 7A, CDK5RAP3 deficiency forced the fluorescence brightness to increase, and it is more concentrated around the cytoplasm near the nucleus compared with the EtOH group (lysosomes are evenly distributed in the cytoplasm). Additionally, the protein of LC3B was also up‐regulated compared with the EtOH group (Figure 7B). Similarly, the protein level of LC3B (LC3‐I and LC3‐II) was significantly up‐regulated compared with the EtOH group (Figure 7C). The above results suggest that CDK5RAP3 deficiency may lead to autophagy (Figure 7A–C). And mTOR pathway is a classical signalling pathway that regulates autophagy. The expression of mTOR and p‐mTOR protein was down‐regulated compared with the EtOH group (Figure 7C). Then, we tested whether mTOR regulated autophagy is involved in apoptosis caused by CDK5RAP3 deficiency. After 24 h of rapamycin treatment, the protein levels of mTOR and p‐mTOR were decreased (Figure S2A), and LC3‐I and LC3‐II were increased (Figure S2A). In addition, apoptosis‐related protein Bax was up‐regulated (Figure S2A), and anti‐apoptosis‐related protein Bcl2 was down‐regulated (Figure S2A). Flow cytometry results showed that the percentage of live cells was down‐regulated from 97.5% to 92.8% (Figure 7D), and the percentage of apoptotic cells (necrosis, late apoptosis, early apoptosis cells) increased after mTOR and p‐mTOR was decreased (Figure 7D). In conclusion, CDK5RAP3 deficiency inhibits autophagy regulated by the mTOR/p‐mTOR pathway and leads to apoptosis.

### 3.8 The interaction of CDK5RAP3 and RPL26 regulates the mTOR pathway to promote autophagy and lead to apoptosis

**FIGURE 8:**
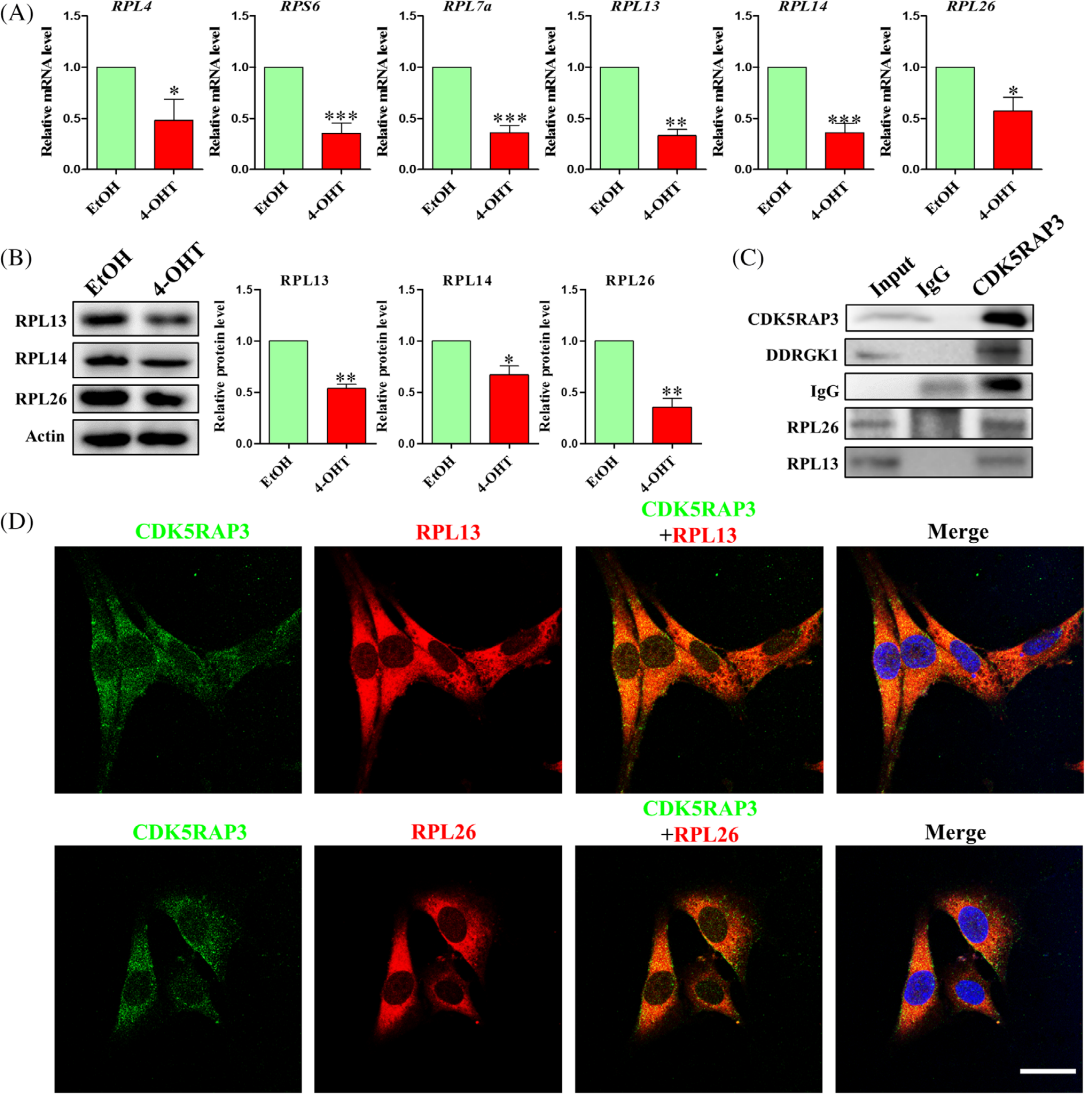
CDK5RAP3 deficiency down‐regulated ribosome relative protein and interact with RPL13 and RPL26. (A) Relative mRNA expression level of *RPL4, RPS6, RPL7a, RPL13, RPL14, RPL26* in EtOH group and 4‐OHT group. (B) Representative WB and relative densitometry analysis of RPL13, RPL14 and RPL26 in EtOH group and 4‐OHT group. (B) The immunofluorescence with CDK5RAP3‐FITC, RPL13‐CY3, RPL14‐CY3 and Hoechst 33342 in MEFs. Scale bar = 10 μm. The results are presented as mean ± SD. *p < 0.05; **p < 0.01

Membrane‐bound ribosomes are located in the endoplasmic reticulum.^42^, ^43^ CDK5RAP3 has been reported as a subtractive interaction with DDRGK1 in UFM1 system.^33^, ^44^ As a discovered ribosomal protein, RPL26 provides a functional link between UFMylation and ER protein homoeostasis.^29^, ^34^, ^35^ We wonder whether there is a direct connection between CDK5RAP3 deficiency and ribosomal function. The mRNA level of ribosome‐related genes including *RPL4, RPS6, RPL7a, RPL13, RPL14* and *RPL26* were significantly down‐regulated with CDK5RAP3 deficiency in MEFs (Figure 8A). Similarly, the protein levels of ribosome‐related genes including RPL13, RPL14 and RPL26 were all down‐regulated accompanied by CDK5RAP3 deficiency (Figure 8B). CDK5RAP3 directly combines with DDRGK1, RPL13 and RPL26 (Figure 8C). Additionally, CDK5RAP3 was co‐localized with RPL13 and RPL26 by cellular immunofluorescence, which showed that they were localized to the cytoplasm (Figure 8D).

**FIGURE 9:**
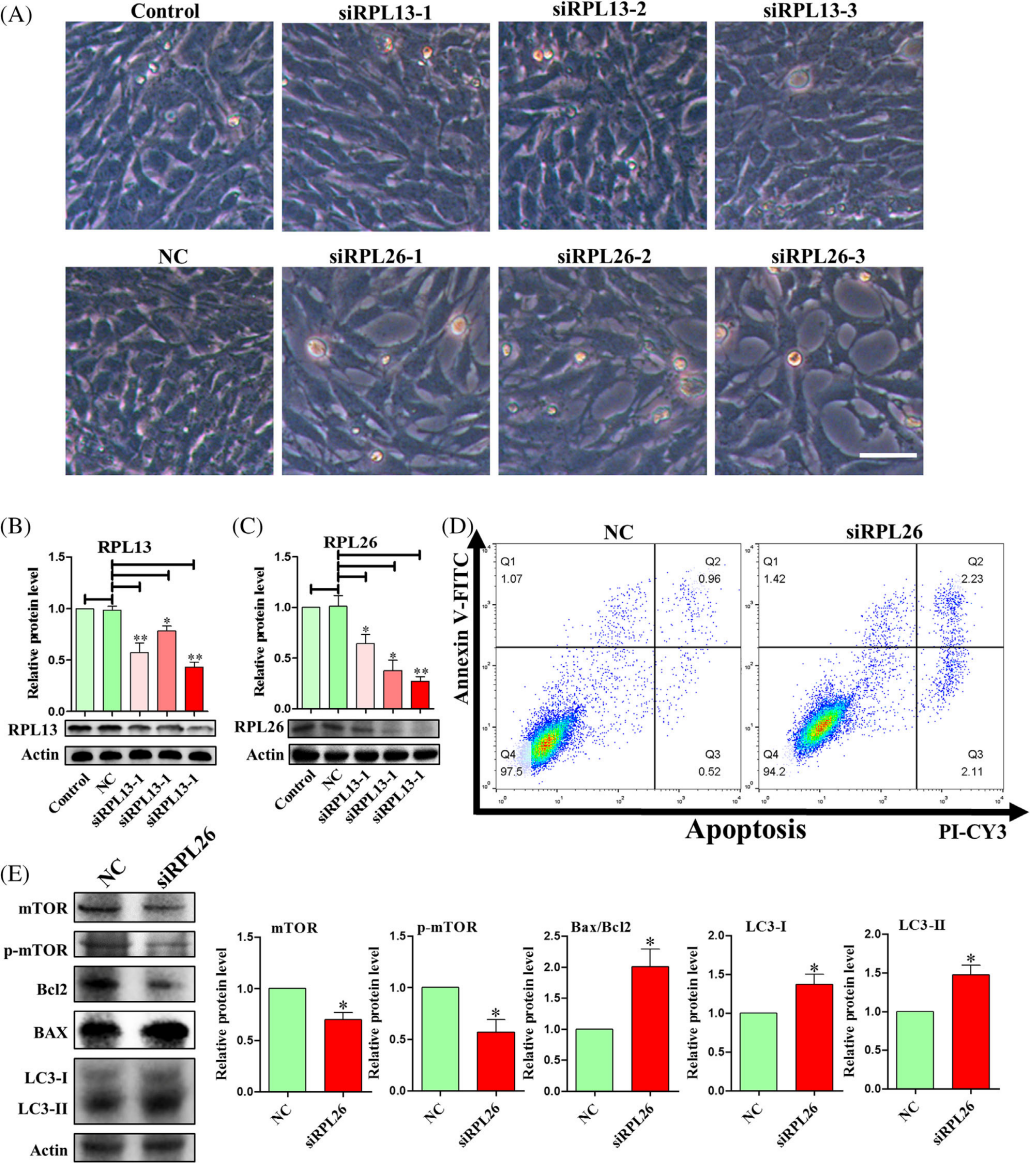
CDK5RAP3 deficiency down‐regulated ribosome relative protein and interact with RPL13 and RPL26. (A) Observe the morphology of MEFs in control group and transfection with nonsense control (NC), siRPL13‐1, siRPL13‐2, siRPL13‐3, siRPL26‐1, siRPL26‐2, siRPL26‐3 groups after 72 h. (B) Representative WB and relative densitometry analysis of RPL13 in control group and transfection with nonsense control (NC), siRPL13‐1, siRPL13‐2, siRPL13‐3 groups. (C) Representative WB and relative densitometry analysis of RPL13 in control group and transfection with nonsense control, siRPL26‐1, siRPL26‐2, siRPL26‐3 groups. (D) Flow cytometry analysis of apoptosis in NC and siRPL26 group. (E) Representative WB and relative densitometry analysis of mTOR, p‐mTOR, Bax, Bcl2, LC3‐I and LC3‐II in NC and siRPL26 group. The results are presented as mean ± SD. *p < 0.05; **p < 0.01

To further confirm the role of RPL13 and RPL26, sequence‐specific siRNA against RPL13 and RPL26 were introduced (Table S3). The cell growth morphology observed under the microscope is shown in Figure 9A. There was no difference in cell growth between the NC (Nonsense siRNA control) group and the control group. As shown in Figure 9A, the siRPL13 group (siRPL13‐1, siRPL13‐2 and siRPL13‐3) was indistinguishable from the control group. SiRPL26 group (siRPL26‐1, siRPL26‐2 and siRPL26‐3) was all less dense compared with the control group. The target protein levels of the siRPL13 group (siRPL13‐1, siRPL13‐2 and siRPL13‐3) and siRPL26 group (siRPL26‐1, siRPL26‐2 and siRPL26‐3) were all decreased compared with the NC group (Figure 9B,C). The above results suggested that RPL26 might be the gene that interacts with CDK5RAP3 and affect the biological functions of cells. SiRPL26‐3 (referred to as siRPL26) was selected for further research. The results of flow cytometry analysis showed that the percent of apoptotic cells increased after the reduction of RPL26 protein (Figure 9D), and the apoptosis‐related protein Bax was up‐regulated, and the anti‐apoptosis‐related protein Bcl2 was down‐regulated. In addition, the protein levels of mTOR and p‐mTOR were down‐regulated, whereas LC3‐I and LC3‐II were both up‐regulated (Figure 9E). The results of cell cycle by flow cytometry showed that the increased percentage of RPL26 significantly reduced the proportion of MEF in the S phase (1.78%), and partially blocked cells in G_2_/M phase (Figure S3B). The above results indicate that RPL26 induces the increase of autophagy by inhibiting the mTOR pathway, which leads to the occurrence of cell apoptosis. These phenomena completely correspond to the effects of CDK5RAP3 deficiency on autophagy and apoptosis.

## 4 DISCUSSION

**FIGURE 10:**
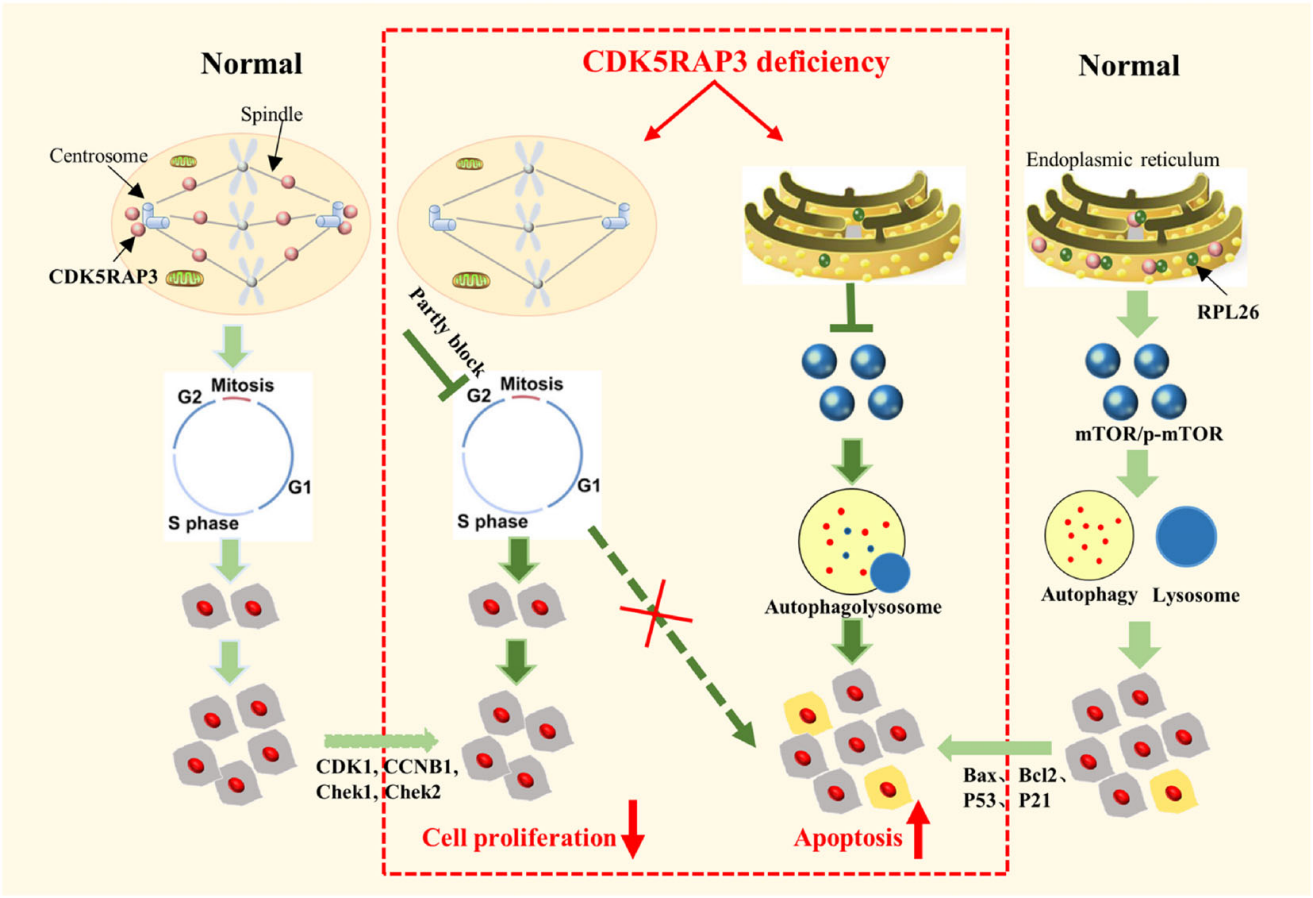
Schematic model for the effect of CDK5RAP3 deficiency on cell growth. In normal, CDK5RAP3 is distributed in the centrosome, spindle and endoplasmic reticulum, the cells undergoes the growth and proliferation. However, when CDK5RAP3 is deficiency, the cell cycle is blocked in G_2_/M and cell proliferation slows down, and the partial cycle block does not cause apoptosis. Additionally, CDK5RAP3 distributed in the endoplasmic reticulum combined with the deficiency of RPL26 will inhibit the mTOR pathway, aggravate autophagy and trigger apoptosis

In this article, we demonstrated that CDK5RAP3 deficiency slows down cell growth by partially blocking the cell cycle and interacting with RPL26, which leads to apoptosis and regulates autophagy by reducing the mTOR/p‐mTOR signalling pathway (Figure 10). CDK5RAP3 has been shown to be important in the early development of Zebrafish epiboly, liver development in mice, and intestinal Paneth cell growth and maintenance in mice.^13^, ^18^, ^45^ CDK5RAP3 deficiency in early embryonic cells causes a G_2_/M arrest, preventing embryos from initiating epiboly.^13^ CDK5RAP3 interacts with UFL1 and serves as a novel substrate adaptor for UBL modification, and CDK5RAP3 deficiency causes ER stress and activates PERK and IRE1α signalling pathways, which lead to normal liver development.^16^ Additionally, CDK5RAP3 has also been shown to promote cell proliferation, and cell cycle‐related regulators, such as CDK1, CCNB1, CCND1, Chek1, Chek2.^7^, ^12^ CDK5RAP3 plays an important role in organism development by regulating cell cycle progression. However, low‐ or high‐level expression makes it controversial that whether CDK5RAP3 is a tumour activator or an inhibitor. In our research, when the cell cycle was disrupted, cell proliferation and growth were inhibited (Figures 1, 2, 3).

The centrosome is the cell's microtubule nucleating core, and during mitosis, the centrosome complex drags the spindle to the poles.^42^ The interaction of CDK5RAP3 with TUBG1 suggests that the CDK5RAP3 protein on the centrosome could be a component of the centrosome complex.^20^ Our results indicate for the first time that CDK5RAP3 is not only exists in the centrosome but also binds to the spindle. The fraction of CDK5RAP3‐FITC bound with the spindle (β‐tubulin‐CY3) in metaphase is shown in Figure 4. When the overflow ring separates the two cells at the end, the overlap (yellow) of CDK5RAP3‐FITC and the microtubule (β‐tubulin‐CY3) in the microtubule fracture area is still visible (Figure 4). CDK5RAP3 deficiency in the centrosome and spindle partially blocks the cell cycle in the G_2_/M phase of mitosis (Figures 3 and 4). The co‐localization of CDK5RAP3 and centrosome proteins suggests that CDK5RAP3 may function as a centrosome complex. The spindle does not always appear, it only appears when the cell undergoes mitosis, and at this time we observed the co‐localization of CDK5RAP3 and the spindle, which suggests that CDK5RAP3 may be either a kinesin or a traction protein to exercise correct guidance spindle alignment and poleward movement. However, we did not observe the localization of CDK5RAP3 in the nucleus after mitosis. How cells regulate the appearance and decrease of CDK5RAP3 remains to be further investigated.

After CDK5RAP3 deficiency, the percentage of G_0_/G_1_ cells was reduced (Figures 3 and 4). The CDK1–CCNB1 complex is one of the primary participants in the S‐ G_2_/M checkpoint, and it is essential for mitosis entry.^46^, ^47^ Furthermore, according to our findings, the CDK5RAP3 deficiency down‐regulated CDK1 and CCNB1 and partially halted the cell cycle in G_2_/M phase (Figure 3). Furthermore, we reported a rise in multinucleated cells (Figures 2E and 3A,B). At the same time, it inhibits cell proliferation and partially affected cell growth (Figure 10). We have found the phenomenon of multinucleated cells in the blood and mammary tumours in the conditional knockout adult mice (data was not shown).

In addition to cell proliferation, apoptosis also has an effect on cell growth. Flow cytometry studies demonstrate that CDK5RAP3 deficiency causes an increase in apoptosis (Figure 6), although the mechanism of apoptosis is unclear. Partially inhibiting the cell cycle at the G_2_/M stage did not result in apoptosis (Figure 7). It was found that Maxer is related to CDK5RAP3 in the endoplasmic reticulum.^14^ According to a plant study, CDK5RAP3 is translocated into transport vesicles when the ER is stressed and defective proteins begin to accumulate.^29^ ER stress is triggered by CDK5RAP3 deficiency.^33^ The co‐localization of CDK5RAP3 and calnexin dual‐cell immunofluorescence staining (yellow) demonstrates that CDK5RAP3 is found in the endoplasmic reticulum (Figure S3A). The homoeostasis of the endoplasmic reticulum ensures protein synthesis and processing, and CDK5RAP3 plays an important role in the maintenance of endoplasmic reticulum homoeostasis. However, CDK5RAP3 deficiency causes lysosome abnormalities (Figure 8A), as well as a surge in autophagy (Figure 8B,C).

Autophagy is a self‐protective mechanism of cells in response to external stress stimuli; however, severe autophagy can trigger cell death patterns.^48^ The role of CDK5RAP3 in autophagy in renal cancer has been identified; however, the mechanism remains unclear.^8^ The mTOR signalling pathway is a well‐known signalling pathway that is involved in autophagy and ubiquitin‐mediated alterations.^49^, ^50^ CDK5RAP3 regulating AKT,^7^, ^9^ GSK3β,^7^ stat3,^25^ MAPK,^27^ NF‐*κ*B,^51^ Wnt^22^ signalling pathway has been studied, but the mTOR signalling pathway has not been studied before. The expression of mTOR and p‐mTOR proteins was suppressed when CDK5RAP3 was deficient. In our study, rapamycin was used to suppress the mTOR and p‐mTOR proteins (Figure 8C), and the downregulation of mTOR/p‐mTOR protein resulted in an increased in the percentage of cells undergoing apoptosis (Figure 8D,E). CDK5RAP3 deficiency also decreased mTOR/p‐mTOR protein levels (Figure 8C) and increased the apoptosis percentage (Figure 5). In conclusion, CDK5RAP3 initiates autophagy and leads to apoptosis by regulating the mTOR signalling pathway.

Additionally, CDK5RAP3 can combine with Ufl1 and DDRGK1 as a Ufmylation adaptor.^16^, ^17^, ^23^ As an autophagy regulator, DDRGK1 is believed to play a key role in modulating lysosomal function.^44^ According to Walczak et al., RPL26 is also the main target of Ufmylation.^35^ It is unclear whether RPL26‐mediated mTOR signalling regulation happens alone or in tandem with CDK5RAP3.^10^ According to our findings (Figure 8A–C), CDK5RAP3 interacts with RPL26, and CDK5RAP3 deficiency reduces RPL26 protein and mRNA levels. In addition, knocking down RPL26 protein expression with interfering RNA reduced mTOR and p‐mTOR protein expression (Figure 9C,E), increased the protein levels of LC3‐I and LC3‐II (Figure 9E) and also increased the percentage of apoptosis (Figure 9D). This indicates that CDK5RAP3 and RPL26 jointly control the mTOR signalling pathway. In our research, CDK5RAP3 was not only localized in the centrosome and spindle with cell cycle progression (Figure 4), but also partially interacted with the ribosomal protein RPL26 localized on the endoplasmic reticulum (Figures 8A and S3A). As a ribosomal protein, we did not find that RPL26 is also expressed in the centrosome or spindle. Therefore, we hypothesized that RPL26 cannot bind CDK5RAP3 in the centrosome or spindle. CDK5RAP3 activates autophagy due to ribosomal arrest during co‐translational protein translocation with ribosomes and leads to the degradation of certain ER proteins.^29^ We found that CDK5RAP3 deficiency caused down‐regulation of RPL26 protein expression, which we believe is the main reason for the induction of autophagy by CDK5RAP3 deficiency. However, the active site requires our further study.

Stable cell growth, including normal cell proliferation, apoptosis and cell differentiation was necessary for tissue growth and organ development. In development, apoptosis was needed in individual fingers from the webbed toe and disappearance of the tail in humans.^52^ In addition, normal proliferation and apoptosis of granulosa cells are important for primordial follicle formation.^53^ However, loss of normal regulation of cell proliferation and apoptosis may lead to tumour or cancer, and CDK5RAP3 was reported related to renal cancer, gastric cancer, colorectal cancer and others.^8^, ^20^, ^21^ We also found that CDK5RAP3 is related to mammary tumours (data not shown). Research on CDK5RAP3 was meaningful for understanding the mechanism of cancer. Our research proved that CDK5RAP3 interacts with RPL26 and maintains the stability of cell growth. CDK5RAP3 deficiency produces partial cell cycle arrest in the G_2_/M phase, but this level of cycle arrest does not result in apoptosis (Figure 10). Exploring the biological function of CDK5RAP3 will help to understand the mechanism of organ development and tumorigenesis related to it, and to understand or treat related diseases according to the mechanism of occurrence as a target gene. In short, CDK5RAP3 absence has two effects on the stability of cell growth. On the one hand, CDK5RAP3 defects associated with the centrosomes and spindles reduce cell proliferation by partially blocking the cell cycle. On the other hand, CDK5RAP3 modulates the mTOR pathway and leads to apoptosis when it interacts with RPL26. However, more research is needed to figure out the reason that CDK5RAP3 defects in the centrosome and spindle partially but not completely prevent the cell cycle. As we mentioned above, the localization of CDK5RAP3 changes dynamically during cell cycle progression. How does this change occur? Does CDK5RAP3 move to the centrosome with tubulin, or does it activate the protein here when the centrosome protein replicates, or is the protein here histone modified? This mechanism needs more research to explore.

## CONFLICT OF INTEREST

The authors declare no conflict of interest.

## AUTHOR CONTRIBUTIONS

All authors participated in the study design revision of the manuscript. Hongchen Yan and Jun‐jie Xu collected the specimens and conducted the experiments, Wei Zhang, Ming Jiang, Guiping Li, Yong Teng analysed data. Hongchen Yan drafted the first version of the manuscript. Hongchen Yan, Ilyas Ali, Guangxun Zhu and Yafei Cai reviewed and commented on the manuscript and approved the final draft.

## Supporting information


**FIGURE S1** The effect of CDK5RAP3 deficiency on mitochondria and Oxidative stress. (A) Flow cytometry analysis of JC‐1 in EtOH group and 4‐OHT group. (B) Flow cytometry analysis of ROS in EtOH group and 4‐OHT group. (C) Flow cytometry analysis of JC‐1 in Scrab group and shRNA group. (D) Flow cytometry analysis of ROS in Scrab group and shRNA group
**FIGURE S2** (A) Representative WB and relative densitometry analysis of Bax, Bcl2, mTOR, p‐mTOR, LC3‐I and LC3‐II in control and rapamycin treated for 24 h
**FIGURE S3** (A) The immunofluorescence with CDK5RAP3‐FITC and Calnexin‐CY3 (endoplasmic reticulum biomarker) in MEFs. (B) Flow cytometry analysis of cell cycle in NC and siRPL26 group. Scale bar = 10 μm

Appendix S1

**TABLE S1** Antibodies used in this paper
**TABLE S2** Primers used for quantitative RT‐PCR
**TABLE S3** Sequences of siRNA oligonucleotides

## Data Availability

All data generated or analyzed during this study are included in this article. Data and material will be made available on reasonable request.
