# Supplementary figures and images for: CDK5RAP3, an essential regulator of checkpoint, interacts with RPL26 and maintains the stability of cell growth
Source: Cell Prolif. 2022 May 4;55(5):e13240. doi: 10.1111/cpr.13240 (PMC9136512; doi:10.1111/cpr.13240)

## Slide 1
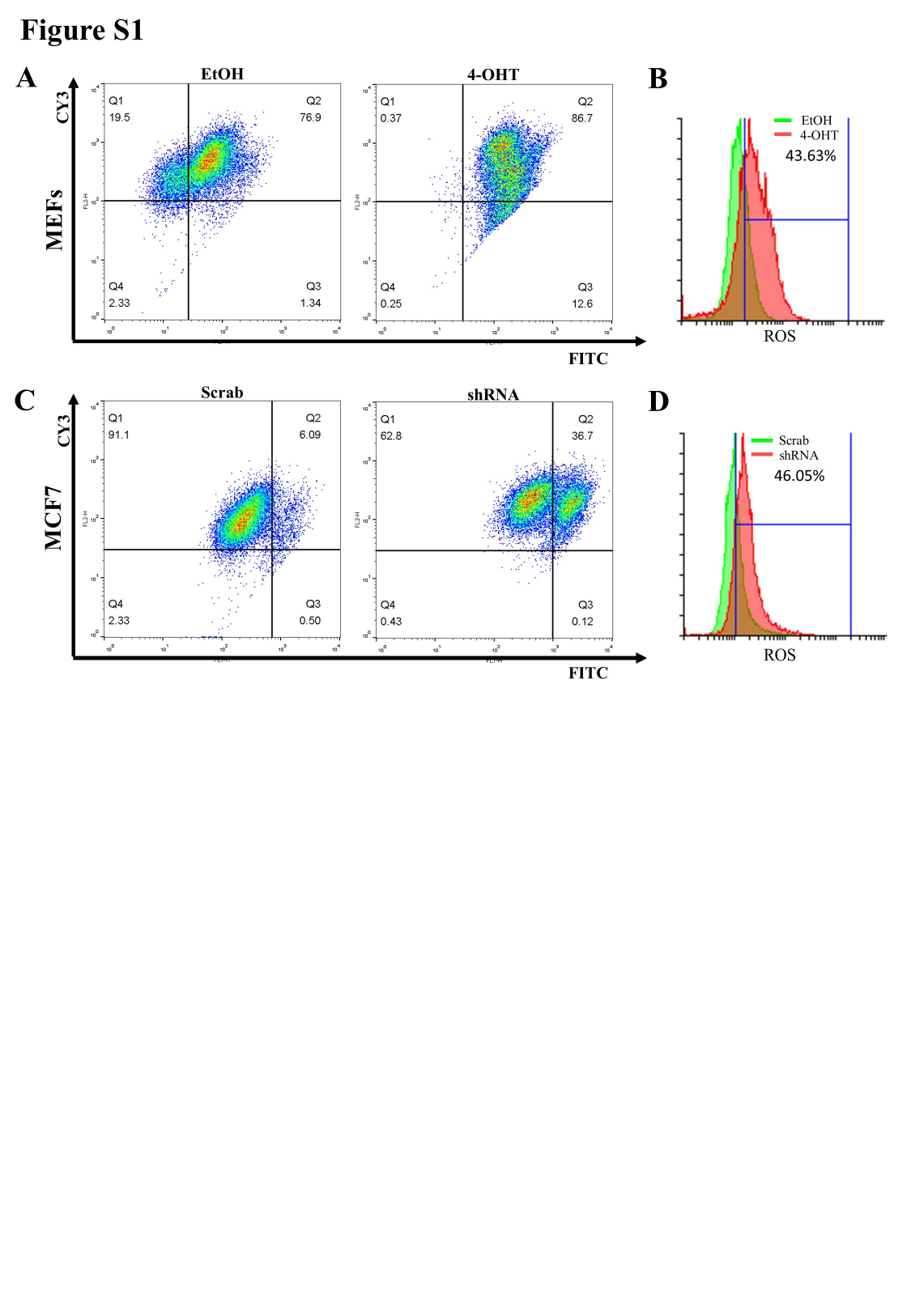

## Slide 2
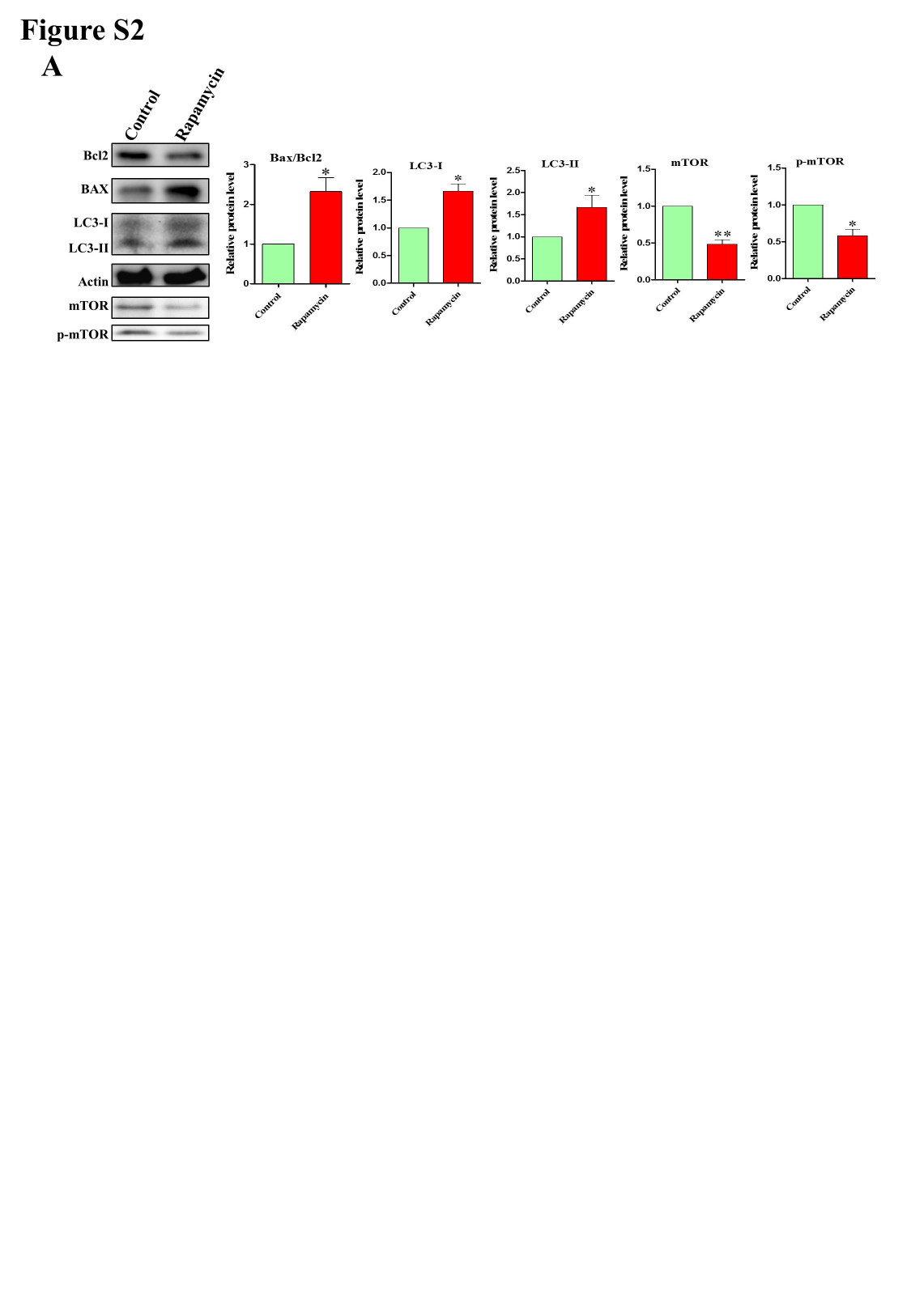

## Slide 3
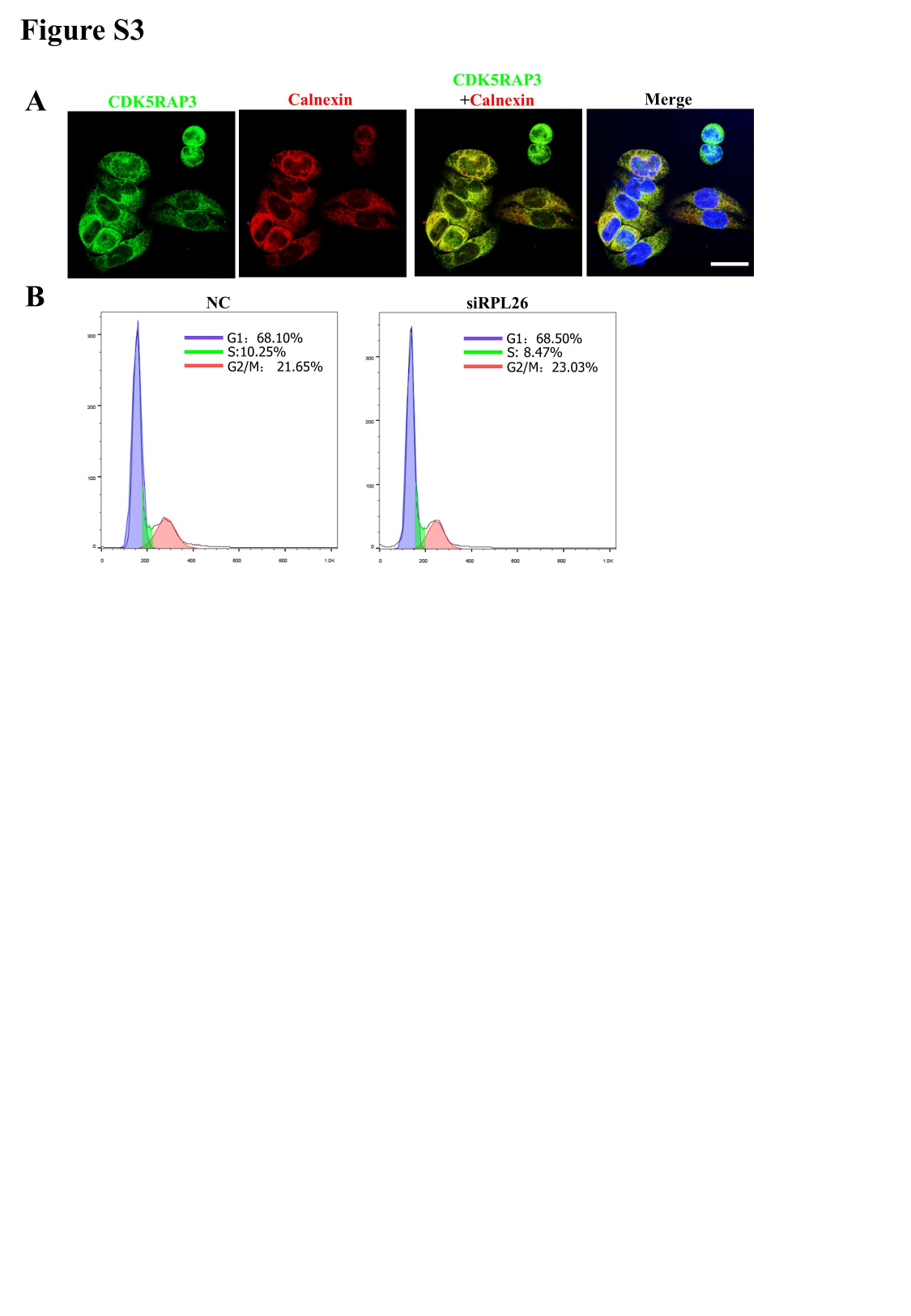

Supplement: Supplementary file 1 — FIGURE S1 The effect of CDK5RAP3 deficiency on mitochondria and Oxidative stress. (A) Flow cytometry analysis of JC‐1 in EtOH group and 4‐OHT group. (B) Flow cytometry analysis of ROS in EtOH group and 4‐OHT group. (C) Flow cytometry analysis of JC‐1 in Scrab group and shRNA group. (D) Flow cytometry analysis of ROS in Scrab group and shRNA group FIGURE S2 (A) Representative WB and relative densitometry analysis of Bax, Bcl2, mTOR, p‐mTOR, LC3‐I and LC3‐II in control and rapamycin treated for 24 h FIGURE S3 (A) The immunofluorescence with CDK5RAP3‐FITC and Calnexin‐CY3 (endoplasmic reticulum biomarker) in MEFs. (B) Flow cytometry analysis of cell cycle in NC and siRPL26 group. Scale bar = 10 μm [file CPR-55-e13240-s003.pptx]
