# Supplementary material for: CDK5RAP3, an essential regulator of checkpoint, interacts with RPL26 and maintains the stability of cell growth
Source: Cell Prolif. 2022 May 4;55(5):e13240. doi: 10.1111/cpr.13240 (PMC9136512; doi:10.1111/cpr.13240)
Supplement: Supplementary file 2 — Appendix S1 [file CPR-55-e13240-s002.docx]

Supplementary Materials and Methods

**Crystal violet staining**

In 60 mm dishes, the number of cells in the first inoculation was the same. Collect samples every 24 h (24 h, 48 h, 72 h, 96 h), and after washing 3 times in PBS, it was fixed in 4% paraformaldehyde for 20 min. Remove paraformaldehyde, cells were washed 3 times with ddH_2_O. Then, stain it for 20 min with 0.1% crystal violet (Macklin, 548-62-9, Shanghai, China). After aspirating the crystal violet and washing it three times with water, let it air dry, then add 2 mL 10% acetic acid to each well and shake for 20 min. At 590 nm, measure and analyze the absorption.

**5-ethynyl-20-deoxyuridine (EDU) proliferation assay**

The cells were grown for an additional 2 h after adding 5-ethynyl-20-deoxyuridine (EDU) (100 mM) (BeyoClickTM EdU-555, C0075S, China) to the 60 mm dishes. The cells were then stained using the following protocol: remove the EDU medium mixture, fix the cells with 4% paraformaldehyde for 30 min at room temperature, wash with glycine (2 mg/mL) for 5 min in a shaker, wash with PBS (add 0.3% Trion X-100 ) twice for 10 min, add reaction buffer (for 1 sample: Click Reaction Buffer (430 μL), CuSO4(20 μL), Azide 555(1 μL) Click Additive Solution (50 μL)) for 30 min in the darkness, wash with 0.5% Triton X-100 for three times, stained with Hoechst (5 mg/mL) for 5 min at room temperature and wash with PBS for 15 min. For immunofluorescence imaging, a confocal microscope was used to photograph and analyze EDU-positive cells.

**Apoptosis detection using the Flow Cytometry**

Annexin V-FITC/PI Apoptosis Detection Kit (Vazyme, A211, China) was performed, according to the manufacturer’s instruction. Digest MEFs and MCF7 with trypsin (Solarbio, T1350, China) without EDTA and incubate the cells with FITC and PI in Binding Buffer for 10 min at room temperature in the dark. Detect with flow cytometer (Becton Dickinson, Mountain View, CA) within 1 h. Data were analyzed using Flow 10 software. Live cells were recorded as a combination of Annexin V-FITC^-^/PI^‑^, the remaining cells were counted as apoptotic cells (early apoptosis cells were recorded as a combination of Annexin V-FITC^+^/PI^-^, and late apoptosis cells were recorded as a combination of Annexin V-FITC ^+^/PI^+^, and necrosis cells were recorded as a combination of Annexin V-FITC ^-^/PI^+^).

**Analysis of cell cycle using Flow Cytometry**

Cell cycle detection kit (Keygentec, KGA511, China) was used for detecting cell cycle. Digest MEFs and MCF7 with trypsin (Solarbio, Beijing, China), wash 3 times in PBS, and fix with pre-cooled 70% ethanol for 20-30 min. After removing the ethanol and washing for three times with PBS, the cells were incubated with Rnase A and PI (1:9) for 30-60 min at room temperature in the dark. Detect with flow cytometer (Becton Dickinson, Mountain View, CA) within 1 h. Flow 10 software was used to analyze the data.

**Use JC-1 to measure mitochondrial membrane potential**

The JC-1 apoptosis assay kit (Keygentec, KGA601, China) was used according to the kit protocol. Take 2 µL of JC-1 (500×) and add in 1 mL of 1× Incubation Buffer, and mix well to obtain 1 mL of JC-1 working solution. Collect the cells, add 1mL JC-1 working solution, cover the cells evenly, and incubate in a 37^o^C, 5% CO_2_ incubator for 15~20 min and detect with flow cytometer within 1 h. Low concentrations are predominantly a monomer (FITC^+^ /CY3^-^), and the dye aggregates yield a red to orange colored emission (FITC^+^/CY3^+^ or FITC^-^/CY3^+^) at high concentrations. When the cell undergoes apoptosis, the mitochondrial transmembrane potential is depolarized, JC-1 is released from the mitochondria, and the intensity of red light is weakened, and it exists in the cytoplasm as a monomer and emits green fluorescence.

**ROS level detection**

Reactive Oxygen Species Assay Kit (Beyotime, S0033, China) was used to detect the ROS level of cells. Dilute DCFH-DA with serum-free culture medium at a ratio of 1:1000 to a final concentration of 10 μM. The cells were collected and suspended in diluted DCFH-DA. Incubate in a 37ºC cell incubator for 20 min. Mix by inversion every 3-5 min to make the probe and cells fully in contact. Wash the cells three times with a serum-free cell culture medium to fully remove the DCFH-DA that has not entered the cells. Detect with flow cytometer at 480 nm.

**COIP**

The r-Protein A/G Magenetc IP/Co-IP kit (ACE, China) was used to Process the samples. Collect the cells in a centrifuge tube, add 0.1% Lysis/Washing Buffer Enhanced, poke the cells with the tip of a 1mL syringe, incubate on ice for 30 min, and centrifuge at 13000g to remove the precipitation. Add the primary antibody CDK5RAP3 and IgG as control, and incubate it overnight at 4°C. Pipette r-Protein A/G Magpoly Beads and wash with Lysis/Washing Buffer, then add the above-mentioned Lysis buffer containing the primary antibody and incubate at room temperature for 2 h. Finally, remove the magnetic beads and add 5X SDS and boil it at 95-100°C for 10 min. The next steps were followed the WB operation.
