# Supplementary material for: CDK5RAP3, an essential regulator of checkpoint, interacts with RPL26 and maintains the stability of cell growth
Source: Cell Prolif. 2022 May 4;55(5):e13240. doi: 10.1111/cpr.13240 (PMC9136512; doi:10.1111/cpr.13240)
Supplement: Supplementary file 3 — TABLE S1 Antibodies used in this paper TABLE S2 Primers used for quantitative RT‐PCR TABLE S3 Sequences of siRNA oligonucleotides [file CPR-55-e13240-s001.docx]

**Table S1** Antibodies used in this paper

| **Primary antibodies** | **Vendor** | **Dilution** | Source |
| --- | --- | --- | --- |
| CDK5RAP3(WB/COIP)  CDK5RAP3(IF)  TUBG1(IF) | Abcam(ab157203)  Abnova(H00080279-M01)  Proteintech( 66320-1-Ig) | 1:1000/1:100  1:200  1:200 | Rabbit  Mouse  Mouse |
| β-tubulin (IF) | Afffnit(AF7011) | 1:200 | Rabbit |
| LC3B( IF/WB) | Proteintech(18725-1-AP) | 1:200/1:1000 | Rabbit |
| RPL13( IF/WB/ COIP) | Sangon(D225652) | 1:200/1:1000/1:1000 | Mouse |
| RPL14( IF/WB)  RPL26( IF/WB/ COIP) | Sangon(D263555)  Proteintech(17619-1-AP) | 1:200/1:1000  1:200/1:1000/1:1000 | Rabbit  Rabbit |
| CDK1(WB) | Sangon(D160158) | 1:1000 | Rabbit |
| CCNB1(WB) | Beyotime(AF6627) | 1:1000 | Rabbit |
| PARP(WB)  Cleaved PARP(WB) | Transgen (HA107)  ABClonal(A19612) | 1:1000  1:1000 | Rabbit  Rabbit |
| Caspase3(WB)  Cleaved-Caspase3  Bax(WB)  Bcl2(WB)  DDRGK1(COIP)  mTOR(WB)  p-mTOR(WB)  Calnexin(IF)  Actin(WB)  IgG(COIP) | ABClonal(A2156)  Afffnit(AF7022)  Proteintech (50599-I-AP)  Wanleibio(WL01556)  Proteintech(21445-1-AP)  Abcam(ab32028)  CST(2974T)  Biogot(BS1438)  Sangon(D110001)  Proteintech (B900610) | 1:1000  1:1000  1:1000  1:1000  1:1000  1:1000  1:1000  1:200  1:1000  1:100 | Rabbit  Rabbit  Rabbit  Rabbit  Rabbit  Rabbit  Rabbit  Rabbit  Rabbit  Rabbit |
| **Secondary antibodies**  CY3-conjugated goat anti-rabbit(IF)  FITC-conjugated goat anti-mouse(IF)  HRP-conjugated goat anti- rabbit IgG(WB) | Beyotime (A0516)  Beyotime (A0568)  Beyotime (A0258) | 1:150  1:150  1:1000 | Goat  Goat  Goat |

**Table S2** Primers used for quantitative RT-PCR

| **Genes** | **Genbanks** | **Forward primer sequences** | **Reverse primer sequences** | **Product Length (bp)** |
| --- | --- | --- | --- | --- |
| *CDK1* | NM_007659.4 | ACCATCGCACTGAGGGAAAG | TCAAAGGGAGTGAGGCAACC | 188 bp |
| *CCNB1* | NM_172301.3 | ACAACGGTGAATGGACACCA | TTATGCCTTTGTCACGGCCT | 233 bp |
| *Chek1* | NM_007691.5 | GCATGACGCAAGCAGGTTTT | ACAGCGACAAGCAGTCCTTT | 81 bp |
| *Chek2* | NM_016681.4 | TAGGTTTAGCGCCACTCCAC | TTCATATCCGACCGCGTGAG | 77 bp |
| *P53* | NM_001127233.1 | AGCTCCCATCACTTCATCCC | TCTTACAGGGTGTGGGGTAG | 397 bp |
| *MDM2* | NM_010786.4 | ACTCTGCTTTGTTAACGGGG | GTATTGCACATTGGCCTGGA | 268 bp |
| *RPL4* | NM_024212.4 | ACAACAGACAGCCCTATGCC | CGTCCCCCACGACACATATT | 160 bp |
| *RPS6*  *RPL7a*  *RPL13* | NM_009096.3  NM_013721.3  NM_016738.5 | TGGATGCATTGTGGACGCTA  AAGGCGACGTCCCAACTAAG  AGAAAGTGGCTCGCACCATC | ATACGTCGGCGTTTGTGTTG  GCACCCTTGTCTTCCGAGTT  AGGATGAGCTTGGAGCGGTA | 282 bp  268 bp  115 bp |
| *RPL14* | NM_025974.2 | CGAAAAGCTTGGGAGAAGGC | GCCTTCTTGGCTGGGACTTT | 284 bp |
| *RPL26* | NM_009080.2 | TCTCTTTCCTTTTGCGGCCA | AATGTGAGAGGGCGCATTGA | 117 bp |
| *Actin* | NM_007393.5 | TCGTGGGCCGCCCTAGGCAC | TGGCCTTAGGGTTCAGGGGGG | 243 bp |
| *Bax* | NM_001291428.2 | CATGGGCTGGACATTGGACT | AAAGTAGGAGAGGAGGCCGT | 137 bp |
| *Bcl2* | NM_000633.3 | CCTATCTGGGCCACAAGTGAA | GCCTGCAGCTTTGTTTCATGG | 119 bp |

**Table S3** Sequences of siRNA oligonucleotides

| **Name** | **Sequences of siRNA(5’-3’)** |
| --- | --- |
| *Nonsense Control* | Sense: UUCUCCGAACGUGUCACGUTT  Anti-sense: ACGUGACACGUUCGGAGAATT |
| *Si RPL13--1* | Sense: CAUCCGGAAUGUGUACAAATT  Anti-sense: UUUGUACACAUUCCGGAUGTT |
| *Si RPL13-2* | Sense: CAGUGAGAUACCACACCAATT  Anti-sense: UUGGUGUGGUAUCUCACUGTT |
| *Si RPL13-3* | Sense: GUGUUGGAGAGCUGCAAUATT  Anti-sense: UAUUGCAGCUCUCCAACACTT |
| *Si RPL26--1* | Sense: GGCAAAUACAAGGAAGAAATT  Anti-sense: UUUCUUCCUUGUAUUUGCCTT |
| *Si RPL26-2* | Sense: GUCCAAGUGUACAGGAAGATT  Anti-sense: UCUUCCUGUACACUUGGACTT |
| *Si RPL26-3* | Sense: GUCGUUAUCACCAGGCUAATT  Anti-sense: UUAGCCUGGUGAUAACGACTT |
